# Unveiling Wound Healing Properties of Biostimulated Walnut Kernel Extracts via Epithelial Mesenchymal Transition: Switching a Nutritional Matrix into a Therapeutic Remedy

**DOI:** 10.3390/antiox14091079

**Published:** 2025-09-02

**Authors:** Riccardo Fedeli, Elia Ranzato, Simona Martinotti, Manuela Giovanna Basilicata, Ludovica Marotta, Marianna Fava, Ilaria Cursaro, Giulio Tremori, Gregorio Bonsignore, Gabriele Carullo, Sandra Gemma, Giovanna Aquino, Pietro Campiglia, Giacomo Pepe, Stefania Butini, Stefano Loppi, Giuseppe Campiani

**Affiliations:** 1 BioAgry Lab, Department of Life Sciences, University of Siena, 53100 Siena, Italy; riccardo.fedeli@unisi.it (R.F.); giulio.tremori@student.unisi.it (G.T.); butini3@unisi.it (S.B.); stefano.loppi@unisi.it (S.L.); giuseppe.campiani@unisi.it (G.C.); 2 Department of Science and Technological Innovation, University of Piemonte Orientale, Viale Teresa Michel 11, 15121 Alessandria, Italy; elia.ranzato@uniupo.it (E.R.); simona.martinotti@uniupo.it (S.M.); gregorio.bonsignore@uniupo.it (G.B.); 3 Department of Advanced Medical and Surgical Sciences, University of Campania “Luigi Vanvitelli”, 80138 Naples, Italy or mbasilicata@unisa.it; 4 TheraFood Research, Department of Biotechnology, Chemistry and Pharmacy, University of Siena, 53100 Siena, Italy; ludovica.marotta@student.unisi.it (L.M.); marianna.fava@student.unisi.it (M.F.); ilaria.cursaro@student.unisi.it (I.C.); 5 AREA Science Park, Laboratorio di Multi-Omica Area Sud (LAAS), 84081 Baronissi, Italy; gaquino@unisa.it; 6 Department of Pharmacy, University of Salerno, 84084 Fisciano, Italy; pcampiglia@unisa.it (P.C.); gipepe@unisa.it (G.P.); 7 NBFC, National Biodiversity Future Center, 90133 Palermo, Italy

**Keywords:** skin repair, EMT, walnuts, biostimulants, green extraction, wood vinegar

## Abstract

Walnuts (*Juglans regia* L.) are recognized for their rich nutritional profile and health-promoting properties. This study investigates the impact of biostimulation, via wood distillate application, on the chemical composition and therapeutic potential of walnut kernels, focusing on their wound healing activity. Biostimulated walnuts showed enhanced levels of phenolic compounds including antioxidants, sugars, and amino acids compared to untreated or agrochemically treated controls. Phytocomplexes extracted from walnut kernels using green methodologies were tested on human keratinocytes (HaCaT), revealing pro-migratory effect, boosted by biostimulation. Molecular analyses demonstrated the activation of epithelial–mesenchymal transition (EMT) pathways, marked by downregulation of epithelial markers and upregulation of mesenchymal genes such as MMP3, MMP9, vimentin, and SMAD3. Furthermore, a synergistic effect was observed between extracts derived from biostimulated kernels and manuka honey in promoting wound closure. These findings suggest that kernels from biostimulated walnuts may serve as functional foods, paving the way for their use in regenerative medicine.

## 1. Introduction

**Figure 1:**
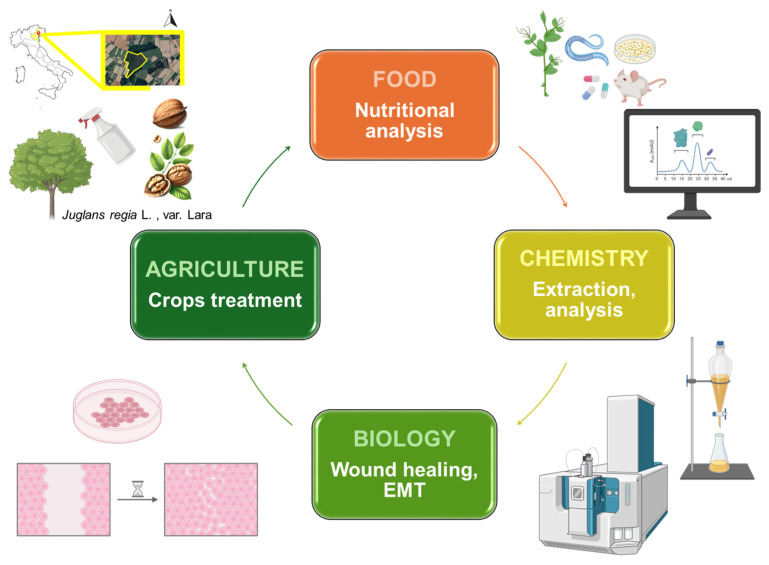
Experimental design of the whole study.

Walnut (*Juglans regia* L.), commonly referred to as Persian or English walnut, is a species belonging to the Juglandaceae family, with a natural distribution ranging from Southeast Europe through the Himalayan region to Southwest China. Walnuts are farmed for commercial purposes and require specific climatic conditions for optimal growth. Walnut trees are generally cultivated as a single tree rather than within mixed woodlands, and warm and sheltered sites are essential for their development, and fertile soils with a pH range of 6.0 to 7.5 are necessary [1]. The fruit is enclosed within a hard shell divided into two compartments, each containing the brown, edible seed (kernel). The nut typically measures 5–7.5 cm in diameter, while the kernel ranges from 1.25 to 5 cm in length [2]. Nut kernels are an important dietary source, and their nutritional and health benefits have been well documented [3]. Walnuts stand out as the whole food with the highest content of α-linolenic acid and present a unique lipid profile that likely plays a role in the positive health effects associated with regular walnut consumption [4]. Notably, walnut kernels represent a nutrient-dense matrix, characterized by elevated levels of unsaturated fatty acids, essential minerals, and polyphenolic compounds and represent a rich source of proteins containing essential amino acids with an elevated nutritional power in comparison to other food matrices and grain proteins [5]. The walnut kernel has long been valued for its rich flavor and nutritional beneficial effects, and is consumed fresh or roasted, or incorporated into a variety of food products. Walnuts also contain significant amounts of sterols, flavonoids, phenolic acids, and pectic substances, which collectively enhance the nutritional profile [6]. In particular, flavonoids and quinones found in the kernels are responsible for their lipid-lowering, antioxidant, antibacterial, chemo-preventive, and liver- and kidney-protective effects [7,8]. From a pharmacological point of view, one of the most interesting properties associated with walnut is the skin-protective effect. Specifically, ethanolic *J. regia* kernel extract exerts a protective effect against the UVC-induced DNA oxidative damage, suggesting a potential antigenotoxic activity [9]. Topical application of walnut root extract significantly accelerated wound contraction and demonstrated beneficial effects on cutaneous wound healing in rats. In particular, the increased collagen synthesis and total protein contents suggested that the extract stimulates cell proliferation. Skin repair was also associated with anti-inflammatory, antioxidant, antifungal and antimicrobial activities [10]. Additionally, the walnut green husk hydroalcoholic extract promoted wound repair in the incision rat model, compared to the control group. The wound repair effect was attributed to the presence of phenolic compounds in the extract, which were responsible for the general anti-inflammatory effect associated with the treatment [11]. Furthermore, *Juglans regia* L. leaf methanol extract was used for topical application in diabetic wounds. At 5% (*w*/*v*) concentration, the extract improved wound closure rate, the volumes of newly formed epidermis and dermis, and the density of fibroblasts and blood vessels. Likewise, the extract increased (i) collagen deposition, (ii) expression levels of transforming growth factor beta (TGF-β) and (iii) vascular endothelial growth factor (VEGF) gene expression, which are the genes appointed for the induction of skin regeneration. Furthermore, this extract reduces the expression levels of tumor necrosis factor alpha (TNF-α) and interleukin-1-beta (IL-1β) genes, which trigger inflammation [12]. Another study outlined the wound healing properties of acetonitrile walnut pellicle and leaf extracts incorporated into silver nanoparticles [13]. Nevertheless, the efficacy of kernel extracts on wound healing has not been investigated yet. Due to their recognized health-beneficial properties, walnuts are receiving increasing attention as a valuable dietary component, thereby boosting their production. In accordance with the European “Farm to Fork” (F2F) strategy, which promotes a transition toward sustainable agriculture and reduced use of synthetic inputs, increasing attention is being devoted to the implementation of environmentally friendly practices in crop cultivation and product development. In this context, the Twelve Principles of Green Chemistry offer a scientifically grounded framework to minimize environmental impact through the use of renewable feedstocks, safer solvents, and energy-efficient processes. The combined application of natural biostimulants and green extraction methods represents a practical embodiment of these concepts, enabling the production of high-value bioactives with therapeutic potential via low-impact, sustainable methodologies [14]. In the recent years, wood distillate, a secondary product generated through the pyrolysis of wood biomass, emerged as a powerful biostimulant, with recent experimental evidence confirming its efficacy in enhancing qualitative, metabolic and nutritional parameters of tomatoes, apples and cowpea [15,16,17]. A graphical representation of the research conducted in this work is reported in Figure 1.

The principles of Green Chemistry have increasingly guided the development of sustainable extraction and agricultural practices. These principles emphasize the reduction in hazardous substances, energy-efficient processes, and the valorization of renewable resources—ideals that are especially relevant in the context of food-derived bioactives and their therapeutic applications. Specifically, we investigated the effect of foliar application of biostimulant wood distillate (0.2%, see specific methodology section) on the chemical profile of walnut (*Juglans regia* L., var. ‘Lara’) kernels, measuring molecular and nutritional parameters. For comparison, we included the untreated controls derived from trees grown exclusively with water and walnuts derived from trees grown with foliar application of commercial and commonly used agrochemicals. Moreover, in this work we investigated for the first time the efficacy and the mechanism of action of biostimulated walnut kernel phytocomplexes in promoting wound healing activity in human keratinocytes, HaCaT cell line. Cutaneous wound healing progresses through distinct but overlapping stages, ultimately aiming to regenerate a stratified epithelium and restore the epidermal barrier. A central step in this process is re-epithelialization, driven by the proliferation and migration of keratinocytes at the wound edges. This tightly regulated, spatiotemporal process is the epithelial-to-mesenchymal transition (EMT) [18]. Accordingly, in order to determine the effect of phytocomplexes on EMT and their efficacy in promoting wound healing, we performed specific biological experiments comparing our results to those obtained with manuka honey (**MH**), a natural food product known to stimulate wound healing and nowadays used as therapeutic remedy (Figure 1). Moreover, we also investigated the synergistic effect on wound healing of **MH** and wood distillate-biostimulated kernel extracts.

## 2. Materials and Methods

### 2.1. Experimental Scheme and Sample Recovery

The study was conducted in 2023 in an experimental orchard located in Cessalto, Treviso, Italy (latitude: 45°42′34″ N, longitude: 12°38′45″ E, altitude: 5 m a.s.l; Figure 1). The experiment involved the species *Juglans regia* L., var. ‘Lara’, planted on 16 February 2017, at a density of 315 plants per hectare. The trees were transplanted mechanically using an MT transplanter, with a row spacing of 4.5 m and an intra-row spacing of 7 m, following a vertical row arrangement. The experimental site is characterized by a loamy silt soil (53% silt, 23% sand, 24% clay), with a pH of 8.2, a CEC of 17.7 meq/100 g, an organic matter content of 1.5%, and a good fertility level. The experimental setup involved three treatment conditions: (i) untreated controls (named “**UTC**”), in trees grown exclusively with water; (ii) the first treatment group (named “**WD**”), with trees receiving foliar applications of 0.2% wood (BioDea [19]); (iii) the second treatment group (named “**AC**”), with trees treated with foliar application of commercial and commonly used agrochemicals. The treatments, performed every 7–10 days from March 2023 until the end of the trial, started after the “Prayer’s Stage” a critical phase of vegetative growth, during which young leaves emerge in a vertical position, resembling hands joined in prayer. This stage marks the onset of photosynthetic activity and plays a key role in canopy development and future yield potential. At the end of the trial (October 2023), walnuts (approx. 200 x plot x treatment) were randomly picked and transported to the laboratory. The experiment was arranged in a randomized complete block design with 3 replications and 3 treatments. Each experimental unit consisted of 4 trees, arranged in plots with a width of 7 m and a length of 18 m, resulting in a total treated plot area of 126 m^2^ per unit. Before the analysis, all the walnuts were opened, and the kernel was homogenized using a professional mixer. The purity of all the reagents was >95%.

### 2.2. Antioxidant Compounds

The total phenolic content (TPC) and total flavonoid content (TFC) were analyzed from walnuts dried in the absence of light, following a published protocol [20]. For the extraction process, 0.5 g of the sample was immersed in 5 mL of 80% methanol (*v*/*v*). The mixture was agitated using an orbital shaker (ASAL VDRL model 711, Cernusco s/N, Milan, Italy) for 30 min and then in the dark at 4 °C. After 48 h, the samples were filtered using Whatman No. 1 filter paper, and the resulting extracts were used for TPC and TFC evaluation. The TPC was assessed via a colorimetric assay (Folin–Ciocalteu) as described [16]. In this assay, 0.125 mL of the extract was mixed with 2 mL of distilled water and 0.125 mL of Folin–Ciocalteu reagent. After a 3 min incubation in the dark, 1.25 mL of a 7% sodium carbonate solution and 1 mL of distilled water were added. The solution was vigorously shaken and incubated in the dark for 90 min. Absorbance was then read at 760 nm using an Agilent UV-Vis 8453 spectrophotometer (Santa Clara, CA, USA). Gallic acid (98%, Thermo Fisher Scientific Inc., Rodano, Milan, Italy) was used as the standard in concentrations ranging from 5 to 300 μg/mL, and results were expressed as mg of gallic acid equivalents per gram of dry extract. TFC was determined using the aluminum chloride colorimetric technique, as described [21]. In this procedure, 0.25 mL of extract was combined with 0.075 mL of 5% NaNO_2_. After 5 min, 0.075 mL of 10% AlCl_3_ was added. The samples were shaken and left in the dark for 5 min before neutralization with 0.5 mL of 1 M NaOH. The mixtures were kept in the dark for an additional 15 min, and absorbance was recorded at 415 nm using the same UV-Vis spectrophotometer. Quercetin (≥95%, Merck KGaA, Darmstadt, Germany) served as the reference compound, with standards ranging from 12.5 to 150 μg/mL. Results were expressed as mg quercetin equivalents per gram of dry extract. Vitamin C content was measured according to a published protocol [22]. Briefly, 200 mg of the samples was homogenized in 0.8 mL of 10% (*w*/*v*) trichloroacetic acid. The homogenate was filtered through gauze, cooled in an ice bath for 5 min, and centrifuged at 3000 rpm for 5 min. A volume of 0.4 mL of the supernatant was then mixed with 1.6 mL of distilled water and 0.2 mL of 0.2 M Folin–Ciocalteu reagent (Carlo Erba, Cornaredo, MI, Italy). After 10 min in dark conditions, absorbance was measured at 760 nm using the Agilent UV-Vis 8453 spectrophotometer. Sample concentrations were calculated using a calibration curve constructed from 0.05–0.2 mL of a 100 μg/mL L-ascorbic acid (BioXtra, ≥99.0%, crystalline) stock solution.

### 2.3. Soluble Sugars and Pectin Content

The quantification of non-structural carbohydrates (sugars and pectin) was carried out based on a previously described method [15]. About 0.5 g of the sample was homogenized in 4 mL of deionized water and centrifuged at 15,000 rpm for 5 min. The resulting supernatant was passed through a 0.45 μm syringe filter before analysis via HPLC (ArcHPLC, Waters), equipped with a Waters 2410 refractive index detector (Milan, Italy). Separation of sugars was achieved using deionized water as the mobile phase, with a flow rate of 0.6 mL/min, and a Waters Sugar-Pak I ion-exchange column (6.5 × 300 mm), maintained at 90 °C with an external column heater (Waters Column Heater Module). Quantitative analysis of sugars and pectin was performed using calibration curves generated from analytical-grade sugars (Merck), dissolved in deionized water at concentrations ranging from 0.1 to 0.5 mg/mL.

### 2.4. Total Soluble Protein and Free Amino Acids Content

The concentration of total soluble proteins was determined following a previously described method [23]. 0.2 g of sample was homogenized in 4 mL of deionized water (dH_2_O) and centrifuged at 4000 rpm for 5 min. From the supernatant, 0.2 mL was combined with 0.8 mL of Bradford reagent (Sigma-Aldrich, Darmstadt, Germany). Absorbance readings were taken at 595 nm using a UV-Vis spectrophotometer (Agilent 8453, Santa Clara, CA, USA). Protein concentration was calculated using a standard curve (ranging from 10 to 100 μg/mL) generated with bovine serum albumin (Sigma-Aldrich, Darmstadt, Germany). The concentration of the free amino acids content was determined following the methods described [24]. The samples were homogenized in 2 mL of deionized water and centrifuged at 4000 rpm. Following the AccQ Tag protocol (Waters, Milford, MA, USA), 10 μL of each reconstituted extract was derivatized with a fluorescent reagent (AQC, Waters, Milford, MA, USA) in the presence of 0.02 M borate buffer to label free amino acids (FAAs). The separation and quantification of FAAs were performed using a HPLC system (LC1, Waters, Milford, MA, USA), equipped with a C18 column (250 × 4.6 mm, 5 μm; Agilent, Santa Clara, CA, USA) maintained at 20 °C, and a scanning fluorescence detector (Waters, Milford, MA, USA), set to an excitation wavelength of 250 nm and emission at 395 nm. The chromatographic separation utilized two mobile phases: (A) a solution consisting of 22.9% (*w*/*v*) sodium acetate in deionized water, 7.7% (*v*/*v*) phosphoric acid, and 4.1% (*v*/*v*) triethylamine; and (B) a 60% (*v*/*v*) acetonitrile solution in deionized water. Amino acid concentrations were quantified by comparing the chromatographic peak areas to those of known standards (WAT088122, Waters, Milford, MA, USA) using Clarity software (DataApex v90A).

### 2.5. Mineral Element Content

Mineral composition was analyzed following a reported procedure, utilizing a portable X-ray fluorescence (XRF) analyzer [25]. Approximately 1 g of dried walnut kernel material was placed in a plastic sample cup and inserted into the instrument’s appropriate chamber. The concentrations of calcium (Ca), copper (Cu), iron (Fe), potassium (K), manganese (Mn), phosphorus (P), sulfur (S), and zinc (Zn) were assessed using the Geochem mode. Each analysis involved three beams, with an acquisition time of 20 s per beam. Method accuracy was confirmed through comparison with 14 certified plant reference materials, as previously described [26]. Mineral concentrations were reported as milligrams of each element per kilogram of dry weight.

### 2.6. Green Extraction of Walnut Samples

For the extraction of bioactive compounds, three different solvent systems were selected based on their efficiency in extracting organic components from walnut samples and their compliance with green chemistry principles. Water (**A**), acetone (**B**), and a “green” Folch mixture (**C**), which is a mixture of ethyl acetate and ethanol in a 2:1 ratio, were selected as extraction solvent systems [27]. For the extraction, 5 g of each triturated walnut sample was weighed and placed in separate containers for maceration with the respective solvent. The samples were left to macerate in the dark for 18 h at room temperature, to ensure adequate extraction of the bioactive compounds. Following the maceration period, the extracts were filtered and transferred into pre-weighed Falcon tubes. The solvents were removed under a stream of nitrogen, and the samples were subsequently freeze-dried. The percentage of extraction yield for each sample was calculated by expressing the mass of the obtained extract as a percentage of the initial walnut sample. This procedure was repeated for all three extraction conditions, and the results were analyzed to determine the extraction efficiency for each walnut sample and solvent combination.

### 2.7. UHPLC-PDA-ESI-Orbitrap-MS/MS Analyses

*Juglans regia* L. extracts were analyzed with a UHPLC-PDA-ESI-Orbitrap-MS/MS system. The UHPLC-HRMS/MS experiments were carried out on a Thermo Scientific™ Vanquish™ UHPLC equipped with a VF-P10-A binary pump, a VH-C10-A column oven, and a VF-A10-A autosampler (Agilent technologies, Santa Clara, CA, USA), coupled online to an Orbitrap Exploris™ 120 mass spectrometer (Thermo Fisher Scientific, Bremen, Germany) fitted with a heated electrospray ionization (HESI-II) source operated in negative mode.

Chromatographic separation employed a Luna^®^ Omega Polar C18 column (1.6 µm, 100 Å, 100 × 2.1 mm; Phenomenex, Bologna, Italy) at 40 °C with a 0.4 mL/min flow rate. The mobile phases were water (A) and acetonitrile (B), both containing 0.1% formic acid. The gradient was programmed as follows: 0.01–2.00 min, 0% B; 2.01–18.00 min, 0–50% B; 18.01–20.00 min, 50–95% B; 20.01–22.00 min, 95% B; 22.01–22.5 min, 95–5% B, followed by 5 min re-equilibration.

Mass calibration was performed in both polarities using Thermo Pierce™ FlexMix™ Calibration Solutions. Data were acquired in full MS (100–1500 *m/z*, 60,000 FWHM) and data-dependent MS/MS (15,000 FWHM) with a normalized collision energy (NCE) of 30. Source parameters were: sheath gas, 40 (a.u.); auxiliary gas, 15 (a.u.); spray voltage, +3.2/−2.8 kV; capillary temperature, 320 °C; auxiliary gas heater, 300 °C.

Peak annotation relied on MS/MS fragmentation matching with spectral libraries, literature data, and external standards when available. Compound identification followed the Metabolomics Standards Initiative (MSI): level 1 (confirmed with reference standards), level 2 (putative by spectral/literature match), and level 3 (putative by chemical class similarity and chemotaxonomy). Data were processed using FreeStyle™ 1.8 SP2 and Compound Discoverer™ 3.1 (Thermo Scientific, San Jose, CA, USA) for baseline correction, noise reduction, spectral alignment, peak detection, and metabolite identification based on exact mass (<5 ppm tolerance), molecular formula, and MS/MS fragmentation [Fragment Ion Search (FISh)] against mzCloud, MassList, and ChemSpider databases.

### 2.8. Semi-Quantitative Analysis

For the semi-quantitative analysis gallic acid, ellagic acid, chlorogenic acid, sinapic acid, (+)-catechin, 4-coumaric acid and oleic acid were used as external standards in negative ionization mode. The calibration curves were obtained in a concentration range of 0.05–200 µg mL^−1^, using five concentration levels with triplicate injections for each level. Linear regression was used to generate the calibration curves with R^2^ values ≥ 0.999. Extracted ion chromatograms (XIC) area of the standard were plotted against corresponding concentrations (µg mL^−1^). Limits of detection (LOD) and quantification (LOQ) were calculated by using the standard deviation (SD) and the slope of the calibration curve, multiplied by 3.3 and 10, respectively. The intra-day precision and accuracy were estimated by analyzing three replicates of standards at three different QC levels: low (LQC), medium (MQC), and high QC (HQC) concentrations. The inter-day precision and accuracy were determined by analyzing the three levels of QC samples on three different runs. Method precision was expressed as the relative standard deviation (RSD) of replicate measurements, while accuracy was evaluated as the ratio between the calculated and theoretical concentrations. The criteria for acceptability of the data included accuracy within 85–115% of nominal values and a precision of within ±15% Relative Standard Deviation (%RSD) (Table S1). The compound contents in the sample were expressed as microgram equivalents per gram of dried weight (µg g^−1^ dw), reported as mean ± deviation standard (*n* = 3) (Table S2).

### 2.9. ^1^H Nuclear Magnetic Resonance (NMR) Investigation

For the NMR analysis, a deuterated liquid–liquid extraction protocol was employed to better characterize the polar and apolar fractions of the extracts. The biphasic system enabled the separation of bioactive compounds based on their polarity, allowing for the acquisition of spectra from each fraction individually and facilitating a more detailed analysis. For this purpose, approximately 70 mg of each sample was weighed and solubilized in a mixture of 700 μL CDCl_3_ and 700 μL of a 1:9 mixture of CD_3_OD and D_2_O. The sample mixture was thoroughly shaken to ensure complete dissolution and homogeneous phase separation. After allowing sufficient time for phase separation, the upper (CD_3_OD/D_2_O) and lower (CDCl_3_) phases were carefully separated and placed into individual tubes for NMR analysis. This approach enabled the analysis of the two distinct phases of the biphasic system. The NMR spectra were recorded for each phase separately, and the data were analyzed to identify and characterize the chemical components of the walnut extracts.

All solvents and standards were obtained from Merck (Milan, Italy). NMR spectra were collected at 298 K on a Bruker AVANCE III spectrometer operating at 400.13 MHz for ^1^H NMR, equipped with a Bruker multinuclear z-gradient direct probehead [28,29,30]. ^1^H spectra were acquired with a spectral width of 6000 Hz and 64,000 data points, corresponding to an acquisition time of 5.5 s. A recycle delay was applied to yield a total acquisition time of 15 s, thereby minimizing relaxation effects [31,32]. The assignment of the resonances was performed by analyzing ^1^H NMR characteristics and the comparison of tests in literature.

### 2.10. Cell Culture

Human spontaneously immortalized HaCaT keratinocytes (RRID:CVCL_0038) were cultured in Dulbecco’s Modified Eagle Medium (DMEM) containing high glucose (4.5 g/L) [33]. This basal medium was supplemented with 200 mM L-glutamine, 10% fetal bovine serum (FBS) (Euroclone, Pero, Italy), and a standard antibiotic solution (penicillin/streptomycin). Cells were maintained at 37 °C in a humidified atmosphere with 5% CO_2_ [34,35]. 

### 2.11. Scratch Wound Assay and Analysis

A scratch wound assay was conducted using confluent monolayers of HaCaT keratinocytes [36]. A sterile 0.1–10 µL pipette tip was employed to create a uniform scratch across the cell monolayer. After removing any detached cells through washing, the cultures were incubated for 24 h in fresh media, with or without the addition of different extracts. Upon completion of the incubation period, cells were fixed with 3.7% formaldehyde for 10 min, followed by staining with 0.1% toluidine blue for 20 min. An inverted microscope (Leica Microsystems, Wetzlar, Germany), equipped with a digital camera, was used to capture images and measure the width of the wound space at both the initial (0 h) and final (24 h) time points. Digital images were analyzed using NIH ImageJ software v5. Wound closure was then quantified by calculating the difference between the initial and final wound widths [37].

### 2.12. Quantitative Reverse Transcriptase PCR (qRT-PCR)

To analyze gene expression, total RNA was extracted from cell samples using the NucleoSpin RNAII Kit (Macherey-Nagel, Düren, Germany). Following extraction, cDNA was synthesized from the RNA template with the Transcriptor First Strand cDNA Synthesis Kit (Roche Diagnostics GmbH, Penzberg, Germany). The resulting cDNA was subjected to qRT-PCR amplification on a CFX384 Real-Time PCR Detection System (Bio-Rad Laboratories, Hercules, CA, USA) using Power Sybr Green Mastermix and KiCqStart^®^ SYBR^®^ Green Primers (see Table 1 for primer details). Relative gene expression levels were determined using the comparative CT (ΔΔCt) method [38].

### 2.13. Statistical Analysis

GraphPad Prism 8 (GraphPad Software, Inc., San Diego, CA, USA) [39] was used to perform statistical analysis for all the data except for the UHPLC-PDA-ESI-Orbitrap-MS/MS. Based on the data, one-way or two-way ANOVAs, followed by an appropriate correction (Dunnett’s multiple comparison test, Tukey’s test) were applied. Statistical details (value of n, test used, *p* value) are reported in the Figure Legends. For the UHPLC-PDA-ESI-Orbitrap-MS/MS results, the data were subjected to ANOVA, and mean differences were assessed using Tukey’s HSD test (Statgraphics Centurion 18, Stat Point Technologies, Inc., Warrenton, VA, USA). Significance was set at *p* < 0.05. The correlation analysis to evaluate the correlation between all features and samples was performed by a heatmap hierarchical clustering was carried out. Hierarchical clustering was applied using a complete linkage clustering method with Pearson distance measurement using the online MetaboAnalyst program 6.0 [40].

## 3. Results and Discussion

### 3.1. Nutritional Profile of Walnuts

**Figure 2:**
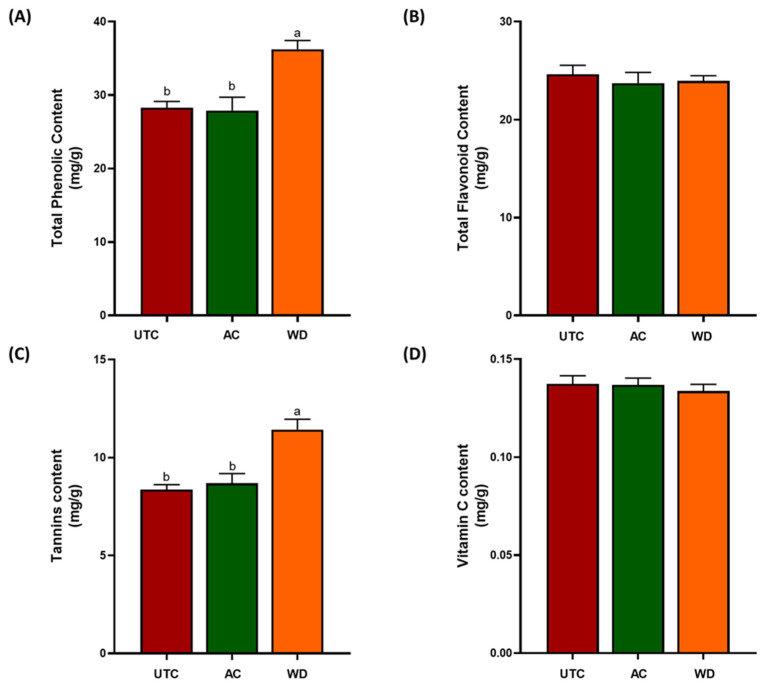
TPC (**A**), TFC (**B**), tannins content (**C**), and vitamin C content (**D**) of kernel, expressed as mean ± standard error. **UTC** = untreated controls; **AC** = samples derived from foliar application of commercial and commonly used agrochemical; **WD** = samples derived from foliar applications of 0.2% wood distillate. Different letters indicate statistically significant differences among the treatments (*p* < 0.05).

The three available kernel samples, i.e., the untreated controls, named **UTC**, the wood distillate-treated named **WD** and the agrochemically treated named **AC**, were subjected to intense studies for the evaluation of nutritional profile. For differently grown walnuts, we performed a series of analysis to evaluate antioxidant profile. Figure 2 reports the data about the measurement of TPC, TFC, tannins and vitamin C levels.

TPC showed a 42.9% increase in **WD** sample compared to **UTC**, while **AC** did not differ significantly from **UTC** (Figure 2A). The three kernels derived from the three treatments showed similar flavonoid levels (Figure 2B). Tannin content was significantly increased in **WD** compared to **UTC** (>31.0%), whereas **AC** did not differ (Figure 2C). In contrast, vitamin C levels are comparable in all samples (Figure 2D). The observed increase in total TPC and tannins in biostimulated walnuts aligns with previous reports suggesting that biostimulants can act as elicitors of secondary metabolite biosynthesis in plants. Phenolic compounds, including tannins, are typically synthesized through the phenylpropanoid pathway, which is often activated in response to environmental stimuli or biotic/abiotic stressors [41].

**Figure 3:**
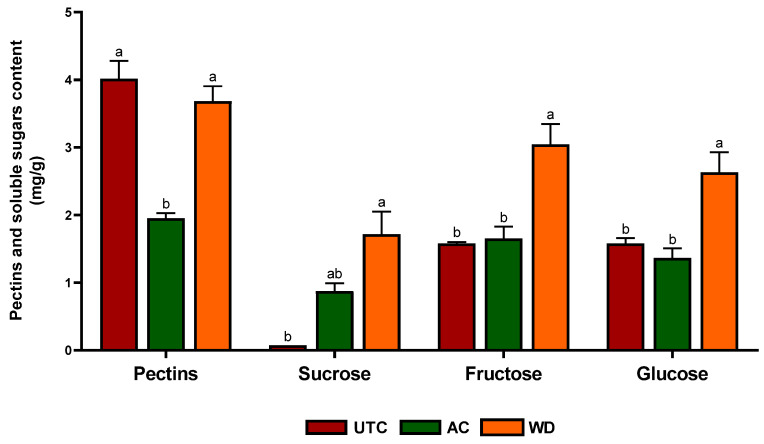
Non-structural carbohydrates (pectins, sucrose, fructose, and glucose (mg/g) of kernel, expressed as mean ± standard error. **UTC** = untreated controls; **AC** = samples derived from foliar application of commercial and commonly used agrochemical; **WD** = samples derived from foliar applications of 0.2% wood distillate. Different letters indicate statistically significant differences among the treatments (*p* < 0.05).

In contrast, the absence of significant changes in TFC suggests that this class of compounds might be regulated differently or require more specific conditions or precursor availability to be modulated. This finding is in line with reports by Gharibi et al. [42] who noted that flavonoid accumulation may depend not only on elicitor presence but also on developmental stage, tissue specificity, and environmental factors such as light intensity. Similarly, the comparable vitamin C levels in the samples indicate that ascorbic acid metabolism may be less sensitive to the external treatments applied, or that it is more tightly regulated due to its crucial role in primary metabolism and redox homeostasis. Vitamin C biosynthesis involves the Smirnoff-Wheeler pathway, which is predominantly regulated by endogenous metabolic cues rather than exogenous stimuli like those provided by wood distillate or agrochemicals [43]. Notably, **AC** treatment did not yield significant differences in any of the antioxidant parameters compared to the **UTC**. This suggests that the synthetic formulation used may have limited or no elicitor-like properties with respect to secondary metabolite biosynthesis. This is an important observation from an agroecological perspective, as it points to the potential of *green*, low-input practices like wood distillate application, to enhance the functional quality of food products without increasing synthetic chemical inputs. Moreover, we analyzed the levels of non-structural carbohydrates such pectin, sucrose, fructose and glucose (Figure 3).

**Figure 4:**
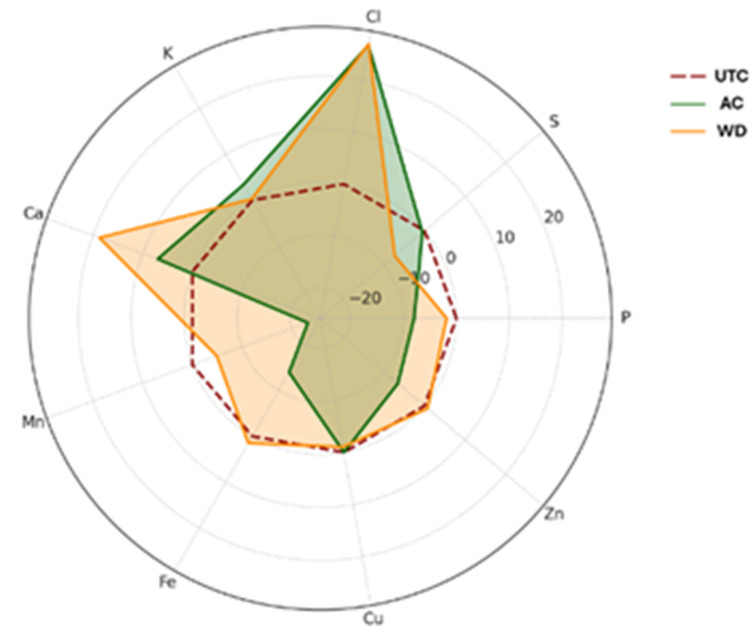
Radar chart of % mineral element content variations. P: phosphorus; S: sulfur; Cl: chlorine; K: potassium; Ca: calcium; Mn: manganese; Fe: iron; Cu: copper; Zn: zinc. **UTC**: untreated control; **AC**: agrochemical; **WD**: wood distillate.

Pectin content was significantly reduced in **AC** compared to **UTC**, with a 35.7% decrease, while **WD** did not differ significantly from **UTC**. Sucrose levels were significantly higher in **WD** compared to both **UTC** and **AC**, showing a 125% increase relative to **UTC**. Similarly, fructose content was significantly increased in **WD**, corresponding to a 66.7% increase, whereas **AC** showed no significant change. Glucose also exhibited a significant increase in **WD**, with a 50.0% rise compared to **UTC**, while no difference was observed in **AC** (Figure 3). The substantial increase in sucrose, fructose, and glucose levels in **WD**-treated samples compared to the **UTC** suggests a stimulatory effect of **WD** on primary carbon metabolism, possibly through modulation of photosynthetic activity or carbohydrate partitioning. These findings align with previous reports which showed that foliar or soil application of wood distillate enhanced sugar accumulation in horticultural crops such as radish (*Raphanus sativus* L.) and tomato (*Solanum lycopersicum* L.), likely due to improved nutrient uptake and metabolic activation [44]. Wood distillate contains a complex mixture of organic compounds, including low-molecular-weight acids, phenolics, and alcohols that may serve as signal molecules, influencing enzymatic pathways involved in sugar metabolism. For instance, acetic acid and small phenolic derivatives can promote root vigor and nutrient assimilation, indirectly supporting enhanced photosynthate production and sugar translocation to fruits and seeds. Various natural biostimulants and elicitors have been reported to influence sugar metabolism by modulating key enzymes such as sucrose phosphate synthase and invertase, which play central roles in sucrose synthesis and breakdown [45]. This regulatory effect on enzymatic activity has been associated with increased sugar accumulation in several crops [46] and may help explain the elevated levels of sugars observed in our wood distillate-treated samples [45]. Interestingly, **AC** did not lead to significant modifications in sugar content and even caused followed by a significant reduction in pectin. This could reflect a mild phytotoxic or stress-related response associated with synthetic fertilizer inputs, as excessive application of synthetic fertilizers has been shown to increase plant yield at the expense of soluble sugar levels and potentially impair cell wall metabolism [47]. Pectin, a major component of primary cell walls, is typically degraded during ripening and stress responses via pectin methyl esterase and polygalacturonase activity [48]. The reduction in pectin under **AC** may be indicative of altered ripening signals or premature cell wall breakdown, which did not occur in **WD** sample. The unchanged pectin levels in **WD** may suggest a more balanced effect on cell wall metabolism, potentially preserving fruit firmness or post-harvest quality. This observation could be of agronomic interest, as maintaining pectin content during development can influence the texture and storage potential of walnut kernels. Additionally, we measured mineral elements to investigate the role of biostimulation on walnuts. Table 2 reports the data about a panel of significant elements. Differences were observed among treatments for several parameters, and a schematic representation is reported in Figure 4.

Phosphorus content was significantly lower in **AC** compared to **UTC**, corresponding to a 7.98% decrease, while **WD** did not differ significantly from **UTC**. Sulfur levels were significantly reduced in **WD** compared to **UTC**, showing a 7.35% decrease, whereas no significant difference was found between **AC** and **UTC**. Potassium content was significantly higher in **AC** than in **UTC**, with a 3.36% increase, while **WD** did not significantly differ from **UTC**. Calcium content showed a marked increase in **WD**, with an 18.7% increase compared to **UTC**; **AC** also presented a significant 7.0% increase relative to **UTC**. Manganese levels were significantly lower in **AC** compared to **UTC**, corresponding to a 23.2% decrease, while **WD** did not differ significantly from **UTC**. Similarly, iron content was significantly reduced in **AC** compared to **UTC**, with a 13.9% decrease; **WD** showed no significant change. Copper levels did not differ significantly among treatments. Finally, zinc content was significantly lower in **AC** compared to **UTC**, showing a 6.56% decrease, whereas **WD** did not differ significantly from **UTC** (Table 2 and Figure 4).

**Figure 5:**
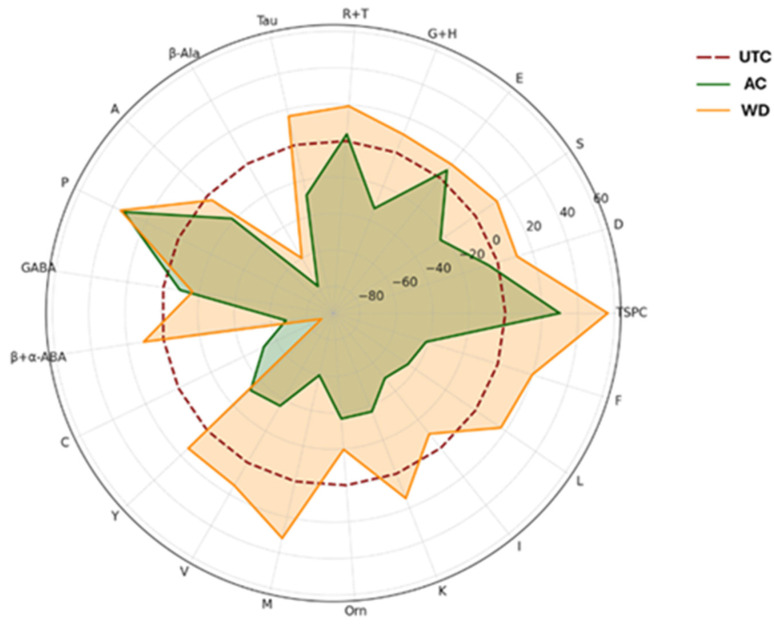
Radar chart of total soluble proteins and free amino acids % variations. TSPC: total soluble protein content; D: aspartic acid; S: serine; E: glutamic acid; C: cysteine; Y: tyrosine; V: valine; M: methionine; K: lysine; I: isoleucine; L: leucine; F: phenylalanine; A: alanine; P: proline; R: arginine; T: threonine; G: glycine; H: histidine; GABA: γ-aminobutyric acid; β-Ala: β-alanine; β + α-ABA: β-aminobutyric acid + α-aminobutyric acid; Tau: taurine; Orn: ornithine; G + H: glycine + histidine; R + T: arginine + threonine. **UTC**: untreated control; **AC**: agrochemical-treated; **WD**: wood distillate-treated.

The significant decrease in P content under **AC** may suggest interference with P uptake or availability in the rhizosphere. Phosphorus is critical for energy transfer, membrane integrity, and nucleic acid synthesis [49]. Synthetic agrochemicals, especially those with high ionic strength or containing nitrogenous compounds, can disrupt P solubility or promote antagonistic ion interactions in soil [50]. In contrast, wood distillate-treated samples, maintained P levels like **UTC**, possibly due to the chelating properties of organic acids present in **WD**, which have been shown to enhance nutrient solubilization and mobility in soil matrices [51]. Calcium levels were markedly elevated in **WD**, more than double the increase observed in **AC**. This is a key outcome, as Ca plays key structural and signaling roles in plant cells. Previous studies on leafy greens treated with **WD** have reported similar increases in Ca and Mg content, possibly due to improved cation exchange dynamics in the rhizosphere or increased expression of Ca transporters induced by organic acids in the **WD** [52]. The ability of **WD** to enhance Ca content may also positively impact walnut firmness, cell wall stability, and post-harvest quality. Interestingly, K levels increased only in **AC**-treated walnuts, while **WD** showed no significant difference with respect to **UTC**. This may reflect the immediate availability of K^+^ in the synthetic formulation, whereas **WD**’s impact on K may be more indirect or slower to manifest. The consistent reduction in key micronutrients such as Mn, Fe, and Zn in **AC**-treated samples is particularly concerning. These elements are central to enzyme function, photosynthesis, and oxidative stress [53]. Their depletion could compromise plant metabolic efficiency and resilience. Conversely, **WD** maintained levels of these micronutrients comparable to **UTC**, indicating a protective or stabilizing effect on mineral nutrition. This finding aligns with studies where **WD** application improved trace element uptake and root growth, potentially via stimulation of microbial activity and enhanced nutrient bioavailability [54]. sulfur content was the only parameter significantly reduced in **WD** samples, which could be due to its conversion to volatile S compounds or its reallocation during secondary metabolism activation. While this reduction is modest, it may warrant further investigation, particularly under S-limited conditions (Table 2). Furthermore, we measured the levels of soluble proteins and free amino acids (Table 3 and Figure 5).

Total soluble protein content was significantly higher in both **AC** and **WD** compared to **UTC**, showing an increase of 29.9% and 56.3%, respectively (Table 3). Among amino acids, β-aminobutyric acid and α-aminobutyric acid content was significantly reduced in **AC**, with a 68.3% decrease compared to **UTC**, while **WD** did not differ significantly from **UTC**. According to the results obtained for sulfur ion, cysteine levels showed a drastic reduction in **AC**/**WD** samples, with a 52.2% decrease in **AC** and an 87.5% decrease in **WD** compared to **UTC**. Methionine content was also significantly reduced in **AC** (−59.8%) but significantly increased in biostimulated walnuts **WD** (32.0% increase) compared to **UTC**. Leucine showed a 45.0% decrease in **AC**, while it was significantly increased in **WD** (+16.9%) compared to **UTC** (Table 3). Similarly, phenylalanine was significantly lower in **AC** (41.3% decrease) and significantly higher in **WD** (+19.4%) compared to **UTC**. The remaining amino acids did not show statistically significant differences among treatments, although general increasing trends could be observed. In fact, most amino acids tend to decrease in **AC** and increase in **WD** compared to **UTC**, with noticeable changes in compounds such as taurine, proline, valine, and lysine (Table 3). The significant increase in total soluble protein observed in both **AC** and **WD** suggests that both treatments stimulate protein synthesis. However, the much larger increase under wood distillate exposure implies a general metabolic stimulation, possibly due to enhanced nutrient uptake and improved C/N balance, as previously reported in crops treated with wood distillate [52]. When examining individual FAAs, clear divergences emerged. The marked reduction in β- and α-aminobutyric acid in **AC** samples may reflect stress-induced metabolic disruption, as these non-proteinogenic amino acids are involved in signaling and stress tolerance.

β-Aminobutyric acid, in particular, plays a key role in priming plant defense responses and is often reduced under conditions of metabolic overload or oxidative stress [55]. The preservation of these amino acids in **WD**-treated samples may indicate a more balanced metabolic state. Of particular interest is the divergent behavior of S-containing amino acids. Cysteine content was drastically reduced in both treatments, suggesting a strong impact on thiol metabolism. Cysteine is not only a building block for proteins, but also a precursor for glutathione and other antioxidants [56]. The reduction under **WD** may reflect its channeling into stress-related pathways (e.g., synthesis of glutathione or phytochelatins), consistent with the enhanced antioxidant profile observed in these samples. Methionine, on the other hand, decreased in **AC** but increased significantly in **WD** samples. Methionine is a key methyl group donor (via *S*-adenosylmethionine) and participates in the biosynthesis of ethylene and polyamines [57]. The accumulation of methionine under wood distillate treatment may indicate stimulation of anabolic and signaling pathways, supporting the hypothesis that wood distillate promotes both primary and secondary metabolism. Branched-chain and aromatic amino acids also showed opposing trends. Leucine and phenylalanine were both significantly reduced in **AC** and significantly increased in **WD**. Phenylalanine is the substrate of the phenylpropanoid pathway and its increase under wood distillate correlates with the elevated TPC reported earlier [58] (Table 3 and Figure 5). This suggests that wood distillate not only promotes phenylalanine availability but may also induce phenylalanine ammonia-lyase activity. Leucine, a key energy-regulating amino acid, may be upregulated under wood distillate treatment to support enhanced protein turnover or osmoprotection. Although not all FAAs showed statistically significant changes, clear trends were observed: **AC** tended to reduce the levels of most FAAs, possibly due to oxidative stress, impaired assimilation, or catabolism, while wood distillate treatment (**WD**) tended to increase them, indicating a possible biostimulatory effect on nitrogen metabolism. Amino acids such as proline, taurine, valine, and lysine, all of which play roles in osmotic balance, redox buffering, or growth, showed noticeable positive increasing trends in **WD** samples, reinforcing the idea that wood distillate supports a metabolic profile favorable to stress resilience and nutritional quality.

### 3.2. Preparation of Walnut Phytocomplexes: A Green Approach to Extraction

**Figure 6:**
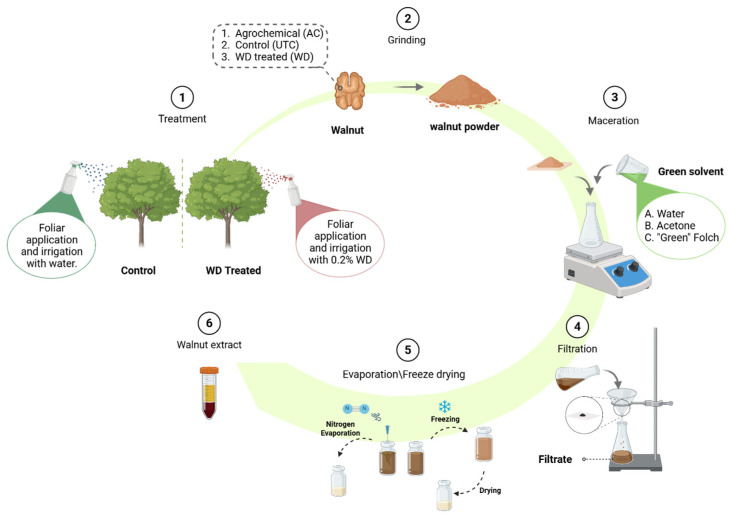
Extraction protocol for the preparation of green extracts. Three different extraction solvents were used to obtain nine different extracts. The extracts were named according to the solvent used for the extraction: water (**A**): **UTC_A**, **AC_A**, **WD_A**; acetone (**B**): **UTC_B**, **AC_B**, **WD_B**; Folch mixture (**C**): **UTC_C**, **AC_C**, **WD_C**.

The data reported in Figure 2, Figure 3, Figure 4 and Figure 5 and Table 2 and Table 3 shed light on the improved nutritional profile of walnuts treated with wood distillate. However, the aim of this work is to evaluate the role of kernels’ extracts on wound repair, by using a recognized cell-based model, i.e., HaCaT keratinocytes. To test the effects of walnut extracts on skin repair, and to evaluate the contribution of biostimulation, we prepared specific phytocomplexes that were previously analyzed at molecular level by LC-MS/MS and ^1^H NMR techniques. To study at the molecular level the contribution of different cultivation methods, we performed three different macerations in three different green solvent systems, i.e., water (**A**), acetone (**B**), “green” Folch mixture (**C**) applying the protocol summarized in Figure 6.

**Figure 7:**
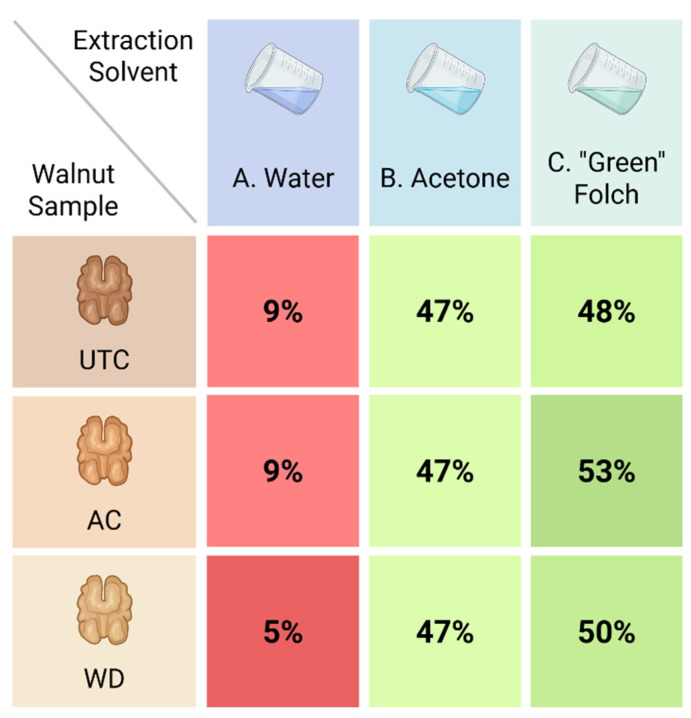
Extraction yields for the different extraction conditions.

The adoption of green extraction methods offers several advantages, including reduced environmental impact, improved operator safety, and the elimination of harmful residues in the final product, making the process more sustainable and aligned with responsible production practices [59]. Nine different extracts were obtained: we conducted the extraction on 5 g of grinded walnuts to obtain: (i) using water (**A**) as extraction solvents: **UTC_A** (0.30 g), **AC_A** (0.31 g), **WD_A** (0.24 g); (ii) using acetone (**B**) for extraction: **UTC_B** (2.58 g), **AC_B** (2.40 g), **WD_B** (2.68 g); (iii) with “Green” Folch mixture (**C**) for extraction: **UTC_C** (2.64 g), **AC_C** (2.77 g), **WD_C** (2.69 g). The extraction yields are summarized in Figure 7.

Under these conditions, water proved to be the least efficient extraction solvent, corroborating literature data that generally favor alternative solvents for the extraction of secondary metabolites from walnuts [60]. Among the other solvents or mixtures tested, acetone exhibited consistent extraction efficiency across all matrices (Figure 7), while the ethyl acetate/ethanol (2:1) Green Folch mixture performed better in the case of **AC** and **WD** walnut samples, although its efficiency remained within the same range as that of acetone.

### 3.3. LC-MS/MS Identification and Quantification

LC-MS/MS analysis of samples obtained from the different nine extracts led to the putative identification of 46 compounds. The results showed substantial variation in both phenol and lipid-derived profiles, depending on walnut origin and extraction method. Representative TIC profiles are shown in Figure S1 while the putatively identified and the corresponding semi-quantitative analysis are reported in Table S2 and Table 4, respectively.

#### 3.3.1. Sinapic and *p*-Coumaric Acid Derivatives

Sinapic acid hexose (peak **16**), detected as a formic acid adduct at *m*/*z* 431, exhibited a fragmentation pattern with product ions at *m*/*z* 385 and *m*/*z* 223, corresponding to the loss of formic acid (46 Da) and a hexose moiety (162 Da), respectively, ultimately yielding the aglycone sinapic acid [61,62,63]. Similarly, *p*-coumaric acid derivative (peak **17**) was also detected as formic acid adduct, showing a molecular ion [M–H]^−^ at *m*/*z* 433. Putative identification was based on the characteristic fragmentation pattern of *p*-coumaric acid, with fragment ions at *m*/*z* 163 and *m*/*z* 119, corresponding to the aglycone and its subsequent breakdown products, respectively [64]. A similar fragmentation pattern was observed for peak **35**, supporting its tentative identification as 3-*p*-coumaroylquinic acid [65].

#### 3.3.2. Gallic Acid Derivatives and Tannins

Peak **19**, with a precursor ion at *m/z* 261, was tentatively identified as a gallic acid derivative based on its MS/MS fragmentation pattern, which showed characteristic fragment ions at *m/z* 169 and 125, corresponding to the typical breakdown of gallic acid [65]. Gallic acid methyl ester (peak **6**) was also detected based on gallic acid fragmentation pattern monitoring the presence of fragment ions at *m/z* 168 and 124. Gallic acid-based tannins like pedunculagin (bis-HHDP-glucose) were tentatively annotated based on molecular ion [M–H]^−^ at *m/z* 783 and on MS^2^ spectra reporting characteristic fragment ions at *m/z* 481, due to the loss of the hexahydroxydiphenoyl (HHDP) moiety, and at *m/z* 301, corresponding to the loss of HHDP-glucose unit. These fragments supported the assignment of peaks **3** and **5** to pedunculagin.

#### 3.3.3. Ellagic Acid Derivatives and Glansreginins

Several ellagic acid-related compounds, such as dicarboxylic acid derivatives (peaks **9**, **10**, **21**, **22**, and **24**), glansreginins (**20**, **27**, **29**, and **30**), ellagic acid pentoside (**23**), and free ellagic acid (**25**) were detected. Dicarboxylic acid derivatives (peaks **9**, **10**, **21**, **22**, and **24**) were characterized by a distinctive fragmentation pattern, consistently producing fragment ions at *m/z* 241 and *m/z* 197 [64]. Notably, compound **27** exhibited a deprotonated molecular ion [M–H]^−^ at *m/z* 592 and yielded MS/MS fragments at *m/z* 403, 343, 241, and 197, leading to its tentative identification as glansreginin A [66]. Isomeric compounds of glansreginin A were also detected and correspond to peaks **29** and **30**, exhibiting analogous fragmentation behavior. In addition, glansreginin B (peak **20**) was identified by its [M–H]^−^ ion at *m/z* 565, showing a similar MS/MS fragmentation pattern with product ions at *m/z* 403, 343, 241, and 197 [66]. Ellagic acid derivatives were identified based on the characteristic fragmentation pattern of ellagic acid, exhibiting typical product ions at *m/z* 257, 229, and 185. Among these, ellagic acid pentoside (peak **23**) was observed with a molecular ion [M–H]^−^ at *m/z* 433, producing free ellagic acid as its main fragment, which was also detected as peak **25**, characterized by a deprotonated molecular ion [M–H]^−^ at *m/z* 300 and confirmed through its MS/MS fragmentation profile [66].

#### 3.3.4. Lipid-Derived Hydroxy Fatty Acids

The 9,12,13-trihydroxy-10,15-octadecadienoic acid (peaks **40** and **44**) was tentatively identified by its deprotonated molecular ion [M–H]^−^ at *m/z* 327. A neutral loss of 98 Da yielded a fragment ion at *m/z* 229, which subsequently underwent dehydration to produce the ion at *m/z* 211 [67]. Similarly, compounds **42** and **43**, exhibiting a deprotonated molecular ion at *m/z* 329, were tentatively assigned as 9,12,13-trihydroxy-10-octadecenoic acid. Their fragmentation involved a neutral loss of 100 Da, generating a fragment ion at *m/z* 229, followed by dehydration to yield the ion at *m/z* 211 [67]. 

### 3.4. ^1^H NMR Analysis

To further assess the chemical composition of the extracts, we carried out ^1^H NMR analyses of all extracts. To fully comprehend the chemical composition, we performed a liquid/liquid extraction on the samples. The extracts were partitioned between CDCl_3_ and D_2_O/CD_3_OD (90:10), to obtain two separate phases that were analyzed by ^1^H NMR. Samples deriving from acetone or Green Folch extractions seem to be similar in their chemical composition, highlighting the similar polarity profile of the extraction solvents (Table 5). From the above-reported experimental setup, a set of amino acids was identified. Isoleucine, identified thanks to the triplet at 0.94 ppm was present only in the extracts obtained from acetone or Green Folch mixture. Other amino acids, like threonine, alanine and arginine were present in all the set of extracts. Of note, leucine was identified in all the samples, except for **UTC_B** and **AC_C**. Additionally, it was possible to identify sucrose, thanks to the -CHOH, a dd at 4.07 ppm [68]. On the other hand, the chloroform fraction of the spectra revealed the presence of fatty acids, identified thanks to different regions of the spectrum as reported in Table 5. In particular, the terminal -CH_3_ typical of the molecules is visible as a triplet at 0.82 ppm, while the characteristic olefinic portion is visible at 5.28 ppm [69].

### 3.5. Scratch Assay Analysis of Different Phytocomplexes—Wound Closure and EMT Activation

**Figure 8:**
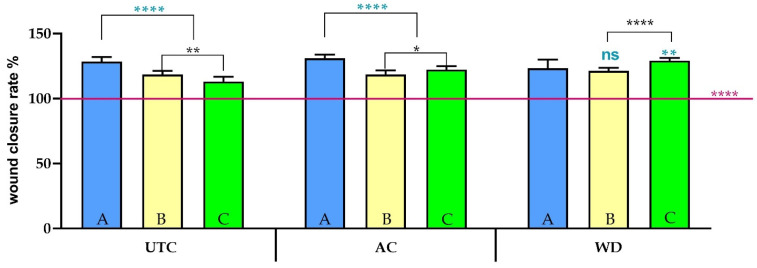
Wound closure rate % in HaCaT cells in control conditions (indicated by the horizontal line in the graph) or exposed to **UTC**, **AC** or **WD** 0.005 mg/mL processed with different solvents (water (**A**), acetone (**B**) and Folch (**C**)) for 24 h, expressed as mean ± SD (*n* = 10). Statistics indicate differences with respect to the control condition (**** *p* < 0.0001, One-way ANOVA followed by Dunnet’s multiple comparison test) or between solvents utilized (with respect to water: **ns** *p* > 0.05, ** *p* < 0.01, **** *p* < 0.0001, Two-way ANOVA followed by Tukey’s test; between acetone and Folch: * *p* < 0.05, ** *p* < 0.01, **** *p* < 0.0001, Two-way ANOVA followed by Tukey’s test).

To evaluate the migratory effects of different extracts, scratch assays were performed on HaCaT keratinocytes. Keratinocyte monolayer scratch assay was used to assess re-epithelialization, which comprises keratinocyte proliferation and migration. Specifically, keratinocytes at the wound site undergo the EMT process, a phenotype change from adherent cells to migratory cells, regulated by several transcription factors. EMT involves a dynamic transformation in which epithelial cells lose their characteristic features—such as cell–cell adhesion, apical–basal polarity, and stable interactions with the extracellular matrix—and adopt mesenchymal properties like increased mobility and invasiveness. To support these changes, EMT-ing cells reprogram their gene expression by suppressing epithelial markers and activating genes that drive cytoskeletal reorganization and promote mesenchymal behavior. In this investigation, we analyzed both wound closure and EMT activation [18]. In detail, the healing of the scratch area was monitored over a period of 24 h. As depicted in Figure 8, the aqueous extracts **UTC_A**, **AC_A** and **WD_A** demonstrated the ability to promote wound repair, with **AC_A** the best performing one. In contrast, the acetone-derived extracts **UTC_B**, **AC_B** and **WD_B** proved to be less effective, showing a limited capacity to enhance scratch closure, and the extent of migration was significantly less pronounced than with **UTC_A**, **AC_A** and **WD_A**. Similarly, the Folch-extracted fractions **UTC_C**, **AC_C** and **WD_C** yielded results comparable to the acetone fraction in terms of promoting wound healing. This outcome is likely attributable to the nature of the Green Folch solution per se.

**Figure 9:**
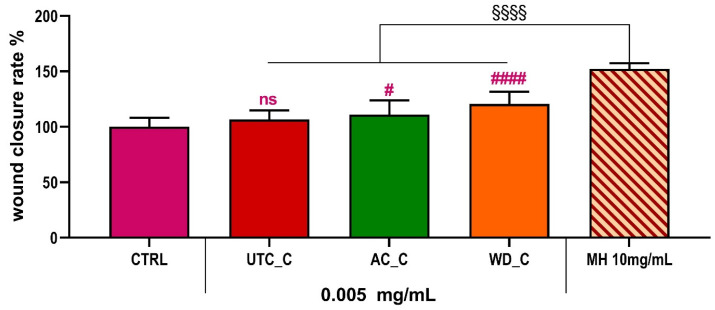
Wound closure rate % in HaCaT cells exposed to **UTC_C**, **AC_C** or **WD_C** for 24 h, expressed as mean ± SD (*n* = 10). Statistics indicate differences with respect to the **CTRL** (ns *p* > 0.05, # *p* < 0.05, #### *p* < 0.0001, One-way ANOVA followed by Dunnet’s multiple comparison test), or with respect to 10mg/mL Manuka Honey (MH, §§§§ *p* < 0.0001, One-way ANOVA followed by Dunnet’s multiple comparison test). CTRL = control condition; **UTC_C**, **AC_C** and **WD_C** as before.

It is a variant of the classical Folch solvent (primarily a chloroform–methanol mixture, specifically designed for the extraction of lipids and highly non-polar compounds), used in green extraction protocols to recover both polar and non-polar compounds from matrices. Given that cell migration and wound healing often involve the action of water-soluble or moderately polar biomolecules (such as certain proteins, peptides, or hydrophilic secondary metabolites) [70], it is plausible that the Green Folch mixture could efficiently recover the primary compounds responsible for the pro-migratory effects observed with their fractions. Specifically, the **WD_C** fraction is the best performing one in terms of wound healing properties, suggesting that this specific phytocomplex contains the substances able to foster wound healing. The efficacy of the Green Folch extract therefore suggests that the key active components driving wound repair are likely hydrophilic or amphiphilic rather than predominantly lipophilic. Considering that **WD_C** showed a relevant pro-migratory effect in the scratch assay, we chose to continue further scratch experiments specifically with the extracts derived from Green Folch extraction. Given the unique composition of Folch extracts, rich in polar and other non-polar compounds, we hypothesized that even subtle pro-migratory activity from this fraction could indicate a novel or previously uncharacterized mechanism of action. Several known pro-migratory factors are hydrophilic; therefore, investigating a less polar fraction could uncover distinct signaling pathways or lipid-mediated effects crucial for wound healing that would be overlooked by focusing solely on aqueous extracts. In particular, the LC-MS data revealed that wound healing effect could be attributed to the presence of a variety of compounds known to promote wound healing. Among the different compounds present, peduncalagin was reported as a modulator of the VEGF receptor, thus promoting re-epithelialization/revascularization [71]. Furthermore, derivatives of vanillin like sinapic acid, have been reported as wound healing agents, promoting cell migration and proliferation by regulating oxidative stress and collagen synthesis [72]. Moreover, ellagic acid has been reported to reduce the expression levels of TNF-α and NF-κB genes, thus limiting the inflammatory process [73]. In line with this, our aim was at comprehensively exploring the potential biological activities of the **UTC_C**, **AC_C**, **WD_C** by analyzing their mechanism of action. By concentrating on the Folch fraction, we aimed to uncover previously uncharacterized contributions to wound repair from the non-polar constituents of walnuts, providing a more comprehensive view of their therapeutic potential. Our analysis (Figure 9) revealed that the **UTC_C** extract did not exhibit a significant difference in promoting wound closure compared to the control group (**CTRL**), which received only DMEM.

This suggests that **UTC_C** has minimal or no direct impact on cell migration in this context. In contrast, both the **AC_C** and **WD_C** extracts demonstrated a significant difference in wound closure rates compared to the **CTRL**. This indicates that **AC_C** and **WD_C** possess properties that actively promote cell migration and, consequently, have the potential to contribute to wound healing. To further contextualize these findings, we also compared the performance of our extracts to that of Manuka honey (**MH**), a known agent with wound healing properties [33]. We tested **MH** at 10 mg/mL.

**Figure 10:**
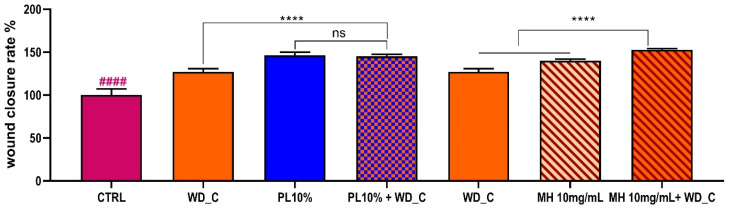
Wound closure rate % in HaCaT cells exposed to **WD_C**, 10% platelet lysate (PL), PL10% + WD, **MH** 10mg/mL, **MH** 10mg/mL + **WD_C** for 24 h, expressed as mean ± SD (*n* = 10). Statistics indicate differences with respect to the CTRL (#### *p* < 0.0001, One-way ANOVA followed by Dunnet’s multiple comparison test), or between single agent and the mix (ns *p* > 0.05, **** *p* < 0.0001, One-way ANOVA followed by Dunnet’s multiple comparison test). **CTRL**, **WD_C** and **MH** as before.

This comparative analysis provided valuable insights into the relative efficacy of our extracts against a recognized standard (Figure 9). Given the promising results observed with the **WD_C** extract in promoting wound closure, we decided to further investigate its synergistic potential with agents commonly used in clinical practice (Figure 10). Therefore, we conducted additional experiments to assess the performance of the **WD_C** extract in combination with platelet lysate (**PL**) [74], a preparation frequently employed in clinical settings for its regenerative medicine properties.

Our findings indicate that while **PL** alone exhibits a very substantial effect on wound closure, the addition of the **WD_C** extract did not result in a notably synergistic action. This suggests that while both compounds are effective, their combined application does not significantly enhance the already potent effect of **PL**.

Conversely, when the **WD_C** extract was combined with 10 mg/mL **MH**, we observed a significantly higher effect on wound closure compared to either the **WD_C** or **MH** used individually. This strong synergistic interaction between the **WD_C** extract and **MH** is especially noteworthy given that the extract is effective at much lower concentrations than honey itself, suggesting their combined application could offer enhanced therapeutic beneficial effects in wound healing.

### 3.6. Molecular Analysis of Epithelial–Mesenchymal Transition and Extracellular Matrix Remodeling

**Figure 11:**
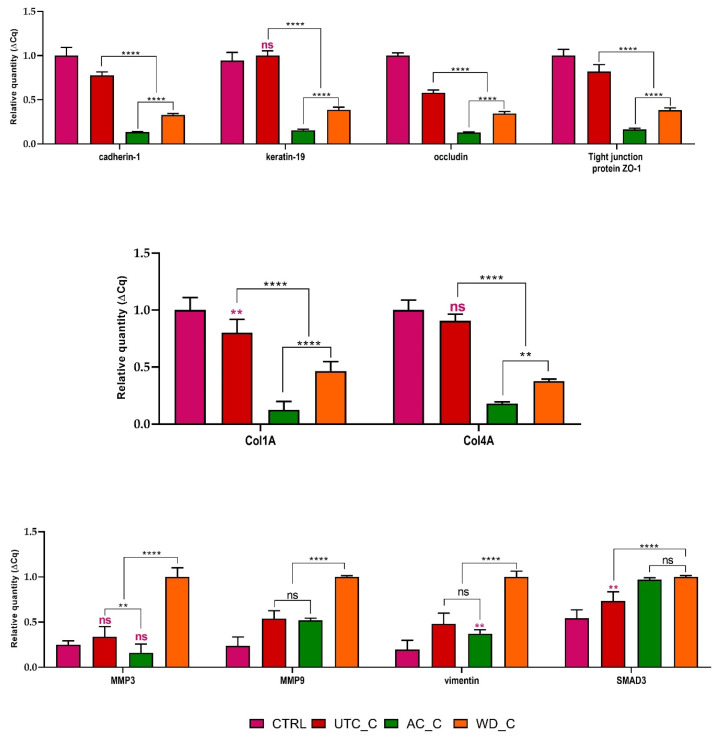
mRNA quantity determined by qRT-PCR of epithelial to mesenchymal transition (EMT) and extracellular matrix (ECM) genes transcripts (cadherin-1, keratin-19, occluding, tight junction protein zonula occludens-1 (ZO-1), collagen 1A and 4A (Col 1A, Col 4A), matrix metalloproteinase 3 and 9 (MMP3, MMP9), vimentin and small mother against decapentaplegic protein 3 (SMAD3)) expressed as mean of relative expression ± SD (*n* = 3). Statistics indicate, for each mRNA group, differences with respect to the **CTRL** (ns *p* > 0.05, ** *p* < 0.01, if not specified *p* < 0.0001, Two-way ANOVA follow by Tukey’s test) or between **UTC_C**, **AC_C**, **WD_C** treatment (ns *p* > 0.05, ** *p* < 0.01, **** *p* < 0.0001, Two-way ANOVA followed by Tukey’s test). **UTC_C**, **AC_C** and **WD_C** as before.

To gain deeper insight into the mechanisms underlying the observed effects of our extracts on wound closure, we performed a quantitative gene expression analysis of genes associated with the epithelial–mesenchymal transition (EMT) reported in Figure 11. EMT is a crucial process in wound healing, involving a shift in cell phenotype that facilitates migration and tissue remodeling [33]. A quantitative gene expression analysis revealed a clear modulation of EMT-related markers in response to the extracts. Specifically, we observed (Figure 11) a significant downregulation of genes characteristic of epithelial cells, including cadherin-1, keratin-19, occludin, and tight junction protein ZO-1 (Zonula Occludens-1).

This decrease in epithelial markers suggests a loss of cell–cell adhesion and epithelial integrity, consistent with cells undergoing EMT. Conversely, our data showed a concomitant and significant upregulation of genes associated with a mesenchymal phenotype. These included matrix metalloproteinase-3 (MMP3), matrix metalloproteinase-9 (MMP9), vimentin, and SMAD3. The increased expression of MMPs indicates enhanced extracellular matrix degradation, a prerequisite for cell migration, while elevated vimentin and SMAD3 levels further confirm a shift towards a migratory, mesenchymal-like cellular state. These molecular findings strongly support the notion that our extracts actively promote EMT, contributing to the observed pro-migratory effects in the scratch wound assay. Furthermore, we observed a notable alteration in the expression of collagens, key components of the extracellular matrix. Specifically, our results indicated a decrease in both collagen I and IV expression. This collagen expression pattern further supports the involvement of EMT, as changes in collagen synthesis and deposition are integral to the dynamic restructuring of the extracellular matrix during this process and subsequent wound healing. These molecular findings strongly support the notion that our extracts actively promote EMT and influence extracellular matrix remodeling, contributing to the observed pro-migratory effects in the scratch wound assay. Our findings demonstrate that our extracts, particularly **WD_C**, possess significant potential in stimulating wound repair, a process intimately linked to the activation of EMT. The scratch wound assay results clearly illustrate the pro-migratory effects of these extracts on cells, with **AC_C** and **WD_C** significantly enhancing wound closure compared to the control. The molecular analyses provide compelling evidence for the underlying mechanism: a distinct shift towards a mesenchymal phenotype. The observed downregulation of epithelial markers (cadherin-1, keratin-19, occludin, tight junction protein ZO-1) coupled with the upregulation of mesenchymal markers (MMP3, MMP9, vimentin, SMAD3) strongly indicates that our extracts are actively inducing EMT. This phenotypic change is crucial for effective wound healing, as it enables epithelial cells at the wound edge to lose their cell–cell contacts, gain migratory capabilities, and invade the wound bed to initiate closure [75]. From a biochemical point of view, some of the recovered metabolites can interact with key mesenchymal markers. In particular, glansreginin A and B seem to modulate the activity of various MMPs, including MMP9 [76], while coumaroylquinic acid seems to modulate the activity of both MMP9 and VEGF receptors, thus promoting anti-inflammatory effects [77]. Furthermore, the alterations in collagen expression, specifically the decrease in collagen I and IV, corroborate the involvement of EMT. This dynamic remodeling of the extracellular matrix is a hallmark of EMT and facilitates cell migration and tissue reconstruction [78,79,80]. The pronounced effects observed with **WD**, both alone and in its synergistic interaction with **MH**, underscore its particular efficacy in driving this beneficial EMT program.

## 4. Conclusions

These results collectively suggest that our extracts, especially **WD_C**, could represent promising therapeutic agents for promoting wound healing through the targeted activation of EMT. Among the various bioactive compounds identified in walnut kernels, phenolics and related antioxidants have gained increasing attention for their ability to modulate oxidative stress, a key factor in tissue repair. The improved antioxidant profile observed in kernels from biostimulant-treated trees may therefore play a pivotal role in promoting re-epithelialization and accelerating wound closure, as suggested by our in vitro findings. Beyond their biological efficacy, the results also highlight the value of applying Green Chemistry principles in both the cultivation and extraction processes. The use of natural biostimulants and green solvents demonstrates that it is possible to develop bioactive formulations with therapeutic potential while adhering to environmentally sustainable methodologies. This work poses the bases for the development of a nutraceutical strategy to treat skin lesions. Nevertheless, further research in vivo will allow us to confirm the migratory role of the extracts.

## Figures and Tables

**Table 1:** List of primers used in qRT-PCR analysis.

Gene	Sequence
cadherin-1	CCGAGAGCTACACGTTCTCTTCAAAATTCACTCTGCC
Keratin-19	AACCATGAGGAGGAAATCAGCATGACCTCATATTGGCTTC
occludin	GGACTGGATCAGGGAATATCATTCTTTATCCAAACGGGAG
ZO-1	TTGTCTTCAAAAACTCCCACGACTCACAGGAATAGCTTTAG
Col1A	GCTATGATGAGAAATCAACCGTCATCTCCATTCTTTCCAGG
Col4A	AAAGGGAGATCAAGGGATAGTCACCTTTTTCTCCAGGTAG
MMP3	GCAGTTAGAGAACATGGAGACGAGAAATAAATTGGTCCC
MMP9	AAGGATGGGAAGTACTGGGCCCAGAGAAGAAGAAAAG
vimentin	GGAAACTAATCTGGATTCACTCCATCTCTAGTTTCAACCGTC
SMAD3	CTACCAGAGAGTAGAGACACTCTCTGGAATATTGCTCTGG
β-actin	TCCCTGGAGAAGAGCTACGAAGCACTGTGTTGGCGTACAG

**Table 2:** Mineral element content (mg/kg) of kernel, expressed as mean ± standard error.

Parameter	UTC	AC	WD
P	2545 ± 33 ^a^	2342 ± 19 ^b^	2498 ± 27 ^a^
S	585 ± 3 ^a^	582 ± 4 ^a^	542 ± 3 ^b^
K	2467 ± 5 ^b^	2550 ± 29 ^a^	2473 ± 7 ^ab^
Ca	530 ± 12 ^b^	567 ± 12 ^b^	629 ± 3 ^a^
Mn	22.4 ± 0.2 ^a^	17.2 ± 0.2 ^b^	21.3 ± 0.4 ^a^
Fe	20.1 ± 0.2 ^a^	17.3 ± 0.3 ^b^	20.4 ± 0.4 ^a^
Cu	9.7 ± 0.3	9.7 ± 0.1	9.6 ± 0.2
Zn	25.9 ± 0.2 ^a^	24.2 ± 0.1 ^b^	26.1 ± 0.2 ^a^

P: phosphorus; S: sulfur; Cl: chlorine; K: potassium; Ca: calcium; Mn: manganese; Fe: iron; Cu: copper; Zn: zinc. **UTC** = untreated controls; **AC** = samples derived from foliar application of commercial and commonly used agrochemical; **WD** = samples derived from foliar applications of 0.2% wood distillate. Different letters indicate statistically significant differences among the treatments (*p* < 0.05).

**Table 3:** Content of total soluble protein (mg/g) and free amino acids (μg/g) of kernel, expressed as mean ± standard error.

Parameter	UTC	AC	WD
Total soluble protein content	8.7 ± 0.2 ^b^	11.3 ± 0.1 ^a^	13.6 ± 0.1 ^a^
Aspartic acid	84.7 ± 8.1	80.2 ± 10.8	94.1 ± 9.1
Serine	35.1 ± 6.6	26.9 ± 4.4	40.1 ± 7.6
Glutamic acid	248 ± 11	262 ± 28	272 ± 17
Glycine + Histidine	34.6 ± 10.3	23.2 ± 2.1	38.3 ± 9.3
Arginine + Threonine	104 ± 8	108 ± 16	124 ± 9
Taurine	27.2 ± 5.5	19.5 ± 3.6	31.6 ± 6.6
β-alanine	40.6 ± 28.2	9.1 ± 3.9	16.3 ± 3.3
Alanine	58.9 ± 11.1	48.1 ± 6.3	56.8 ± 7.3
Proline	69.4 ± 9.6	92.5 ± 27.7	93.9 ± 18.3
γ-aminobutyric acid	64.1 ± 9.2	58.2 ± 8.9	53.7 ± 9.1
β- and α- aminobutyric acid	8.2 ± 3.6 ^a^	2.6 ± 1.6 ^b^	9.1 ± 1.4 ^a^
Cysteine	100 ± 12 ^a^	47.8 ± 5.1 ^b^	12.5 ± 4.4 ^c^
Tyrosine	39.1 ± 11.2	26.4 ± 4.6	44.7 ± 9.9
Valine	35.3 ± 8.6	22.6 ± 3.7	40.4 ± 7.5
Methionine	9.7 ± 3.6 ^a^	3.9 ± 1.8 ^b^	12.8 ± 4.2 ^a^
Ornithine	21.3 ± 4.8	13.5 ± 2.3	17.1 ± 3.9
Lysine	49.9 ± 10.3	31.6 ± 7.1	57.1 ± 9.1
Isoleucine	29.9 ± 10.8	15.2 ± 3.1	26.9 ± 5.5
Leucine	33.1 ± 5.4 ^a^	18.2 ± 3.8 ^b^	38.7 ± 4.1 ^a^
Phenylalanine	24.7 ± 6.0 ^a^	14.5 ± 3.4 ^b^	29.6 ± 6.4 ^a^

**UTC** = untreated controls; **AC** = samples derived from foliar application of commercial and commonly used agrochemical; **WD** = samples derived from foliar applications of 0.2% wood distillate. Different letters indicate statistically significant differences among the treatments (*p* < 0.05).

**Table 4:** Semi-quantitative analysis of *Juglans regia* L. extracts.

**Peak**	**Name**	**Equivalents Expressed**	**UTC_A**		**AC_A**		**WD_A**	
			Concentration * µg g^−1^ dw	**Precision ^#^ (RSD, %)**	Concentration * µg g^−1^ dw	**Precision ^#^ (RSD, %)**	Concentration * µg g^−1^ dw	**Precision ^#^ (RSD, %)**
2	Monogalloyl-glucose	Gallic acid	2.53 ± 0.05 ^b^	1.81%	4.79 ± 0.07 ^a^	1.37%	2.62 ± 0.13 ^b^	4.88%
3	Pedunculagin (bis-HHDP-glucose)	Gallic acid	1.34 ± 0.01 ^c^	0.87%	4.46 ± 0.11 ^a^	2.54%	1.64 ± 0.05 ^b^	3.19%
5	Pedunculagin (bis-HHDP-glucose)	Gallic acid	1.41 ± 0.07 ^b^	4.71%	4.07 ± 0.15 ^a^	3.63%	1.65 ± 0.08 ^b^	4.67%
6	Gallic acid methylester	Gallic acid	4.16 ± 0.03 ^b^	0.70%	11.81 ± 0.09 ^a^	0.77%	2.85 ± 0.07 ^c^	2.29%
8	(+)Catechin	Catechin	0.84 ± 0.01 ^b^	0.87%	10.9 ± 0.04 ^a^	0.34%	0.89 ± 0.01 ^b^	0.83%
9	Dicarboxylic acid derivative 2	Ellagic acid	9.89 ± 0.6 ^b^	6.08%	15.11 ± 0.14 ^a^	0.91%	5.05 ± 0.05 ^c^	0.92%
10	Dicarboxylic acid derivative 3	Ellagic acid	15.05 ± 0.26 ^b^	1.76%	20.33 ± 0.5 ^a^	2.48%	7.35 ± 0.17 ^c^	2.35%
11	Dihydroxytetralone hexoside	Chlorogenic acid	14.62 ± 0.3 ^b^	2.05%	25.46 ± 0.36 ^a^	1.43%	8.24 ± 0.36 ^c^	4.40%
14	HHDP digalloyl glucose	Gallic acid	<LOQ	-	<LOQ	-	<LOQ	-
16	Sinapic acid hexose	Sinapic acid	43.31 ± 0.43 ^c^	0.98%	101.52 ± 0.65 ^a^	0.64%	65.64 ± 0.89 ^b^	1.35%
17	p-Coumaric acid derivative 1	4-Coumaric acid	42.58 ± 1.31 ^c^	3.08%	111.93 ± 0.32 ^a^	0.28%	67.32 ± 0.62 ^b^	0.91%
19	Gallic acid derivative 4	Gallic acid	48.06 ± 1.72 ^b^	3.57%	135.88 ± 2.64 ^a^	1.95%	42.24 ± 0.77 ^c^	1.83%
20	Glansreginin B	Ellagic acid	<LOQ	-	<LOQ	-	<LOQ	-
21	Dicarboxylic acid derivative 3	Ellagic acid	2.74 ± 0.05 ^b^	1.76%	14.4 ± 0.08 ^a^	0.57%	2.55 ± 0.06 ^c^	2.31%
22	Dicarboxylic acid derivative 3	Ellagic acid	2.65 ± 0.07 ^b^	2.68%	15.67 ± 0.56 ^a^	3.56%	2.86 ± 0.1 ^b^	3.39%
23	Ellagic acid pentoside	Ellagic acid	2.35 ± 0.14 ^b^	6.16%	5.27 ± 0.04 ^a^	0.68%	1.13 ± 0.07 ^c^	6.11%
24	Dicarboxylic acid derivative 3	Ellagic acid	50.09 ± 0.46 ^c^	0.92%	122.64 ± 0.31 ^a^	0.26%	51.5 ± 0.41 ^b^	0.79%
25	Ellagic acid	Ellagic acid	48.6 ± 1.2 ^b^	2.46%	88.24 ± 1.83 ^a^	2.07%	40.31 ± 0.95 ^c^	2.36%
27	Glansreginin A	Ellagic acid	194.89 ± 4.7 ^b^	2.41%	281.88 ± 4.04 ^a^	1.43%	202.25 ± 1.31 ^b^	0.65%
29	Glansreginin A	Ellagic acid	12.22 ± 0.11 ^c^	0.94%	69.18 ± 0.15 ^a^	0.22%	14.5 ± 0.15 ^b^	1.02%
30	Glansreginin A	Ellagic acid	8.25 ± 0.18 ^b^	2.14%	11.66 ± 0.03 ^a^	0.24%	7.74 ± 0.06 ^c^	0.71%
35	3-p-Coumaroylquinic acid	4-Coumaric acid	10.7 ± 0.02 ^b^	0.20%	66.89 ± 0.7 ^a^	1.05%	8.61 ± 0.15 ^c^	1.70%
36	5-O-(3′-O-Glucosylcaffeoyl)quinic acid	Chlorogenic acid	2.71 ± 0.06 ^b^	2.08%	7.21 ± 0.15 ^a^	2.05%	2.19 ± 0.05 ^c^	2.17%
40	9,12,13-trihydroxy-10,15-octadecadienoic acid	Oleic acid	8.03 ± 0.18 ^c^	2.24%	47.77 ± 0.13 ^a^	0.27%	9.28 ± 0.31 ^b^	3.31%
42	9,12,13-trihydroxy-10-octadecenoic acid	Oleic acid	92.85 ± 2.1 ^b^	2.27%	173.1 ± 2.61 ^a^	1.51%	<LOQ ^c^	-
43	9,12,13-trihydroxy-10-octadecenoic acid	Oleic acid	2.63 ± 0.17 ^c^	6.43%	9.37 ± 0.05 ^a^	0.52%	3.12 ± 0.09 ^b^	2.95%
44	9,12,13-trihydroxy-10,15-octadecadienoic acid	Oleic acid	2.16 ± 0.06 ^b^	2.90%	5.52 ± 0.18 ^a^	3.22%	1.43 ± 0.04 ^c^	3.07%
**Peak**	**Name**	**Equivalents Expressed**	**UTC_B**		**AC_B**		**WD_B**	
			Concentration * µg g^−1^ dw	**Precision ^#^ (RSD, %)**	Concentration * µg g^−1^ dw	**Precision ^#^ (RSD, %)**	Concentration * µg g^−1^ dw	**Precision ^#^ (RSD, %)**
2	Monogalloyl-glucose	Gallic acid	0.57 ± 0.01 ^a^	1.72%	0.51 ± 0.01 ^b^	2.88%	<LOQ ^c^	-
3	Pedunculagin (bis-HHDP-glucose)	Gallic acid	2.66 ± 0.06 ^a^	2.23%	0.82 ± 0.07 ^b^	8.68%	0.75 ± 0.01 ^b^	1.25%
5	Pedunculagin (bis-HHDP-glucose)	Gallic acid	5.19 ± 0.32 ^a^	6.21%	1.08 ± 0.07 ^c^	6.27%	2.3 ± 0.03 ^b^	1.28%
6	Gallic acid methylester	Gallic acid	3.13 ± 0.04 ^a^	1.25%	0.51 ± 0.01 ^c^	1.88%	0.91 ± 0.01 ^b^	0.85%
8	(+)Catechin	Catechin	0.91 ± 0.01 ^a^	1.58%	0.36 ± 0.00 ^b^	1.27%	0.25 ± 0.00 ^c^	0.84%
9	Dicarboxylic acid derivative 2	Ellagic acid	<LOQ	-	<LOQ	-	<LOQ	-
10	Dicarboxylic acid derivative 3	Ellagic acid	<LOQ	-	<LOQ	-	<LOQ	-
11	Dihydroxytetralone hexoside	Chlorogenic acid	1.61 ± 0.01 ^a^	0.47%	0.75 ± 0.01 ^b^	1.63%	0.35 ± 0.00 ^c^	0.71%
14	HHDP digalloyl glucose	Gallic acid	2 ± 0.09 ^a^	4.52%	<LOQ ^c^	-	0.22 ± 0.01 ^b^	4.30%
16	Sinapic acid hexose	Sinapic acid	8.02 ± 0.1 ^a^	1.28%	5.61 ± 0.12 ^b^	2.20%	3.16 ± 0.19 ^c^	6.08%
17	p-Coumaric acid derivative 1	4-Coumaric acid	5.29 ± 0.18 ^a^	3.38%	4.8 ± 0.04 ^b^	0.78%	3.81 ± 0.22 ^c^	5.67%
19	Gallic acid derivative 4	Gallic acid	2.27 ± 0.03 ^b^	1.25%	2.1 ± 0.04 ^c^	1.92%	2.51 ± 0.00 ^a^	0.17%
20	Glansreginin B	Ellagic acid	2.71 ± 0.07 ^a^	2.55%	2.59 ± 0.04 ^b^	1.47%	0.63 ± 0.01 ^c^	1.34%
21	Dicarboxylic acid derivative 3	Ellagic acid	<LOQ	-	<LOQ	-	<LOQ	-
22	Dicarboxylic acid derivative 3	Ellagic acid	<LOQ	-	<LOQ	-	<LOQ	-
23	Ellagic acid pentoside	Ellagic acid	2.24 ± 0.12 ^a^	5.43%	0.73 ± 0.01 ^c^	1.76%	1.46 ± 0.01 ^b^	0.89%
24	Dicarboxylic acid derivative 3	Ellagic acid	3.37 ± 0.1 ^a^	2.95%	3.5 ± 0.03 ^a^	0.74%	0.24 ± 0.01 ^b^	3.57%
25	Ellagic acid	Ellagic acid	9.55 ± 0.04 ^a^	0.46%	4.69 ± 0.24 ^b^	5.19%	4.02 ± 0.11 ^c^	2.85%
27	Glansreginin A	Ellagic acid	32.44 ± 1.47 ^a^	4.54%	30.75 ± 0.45 ^a^	1.45%	15.24 ± 0.26 ^b^	1.70%
29	Glansreginin A	Ellagic acid	1.35 ± 0.02 ^a^	1.67%	0.88 ± 0.02 ^b^	2.00%	<LOQ ^c^	-
30	Glansreginin A	Ellagic acid	0.94 ± 0.01 ^a^	0.65%	0.94 ± 0.02 ^a^	2.10%	<LOQ ^b^	-
35	3-p-Coumaroylquinic acid	4-Coumaric acid	0.68 ± 0.01 ^b^	1.78%	0.79 ± 0.03 ^a^	3.71%	<LOQ ^c^	-
36	5-O-(3′-O-Glucosylcaffeoyl)quinic acid	Chlorogenic acid	1.53 ± 0.03 ^b^	2.04%	1.68 ± 0.03 ^a^	1.98%	0.44 ± 0.00 ^c^	0.97%
40	9,12,13-trihydroxy-10,15-octadecadienoic acid	Oleic acid	<LOQ	-	<LOQ	-	<LOQ	-
42	9,12,13-trihydroxy-10- octadecenoic acid	Oleic acid	3.94 ± 0.19 ^a^	4.79%	3.43 ± 0.05 ^b^	1.41%	1.23 ± 0.05 ^c^	3.92%
43	9,12,13-trihydroxy-10- octadecenoic acid	Oleic acid	1.34 ± 0.08 ^a^	6.22%	1.4 ± 0.07 ^a^	4.84%	<LOQ ^b^	-
44	9,12,13-trihydroxy-10,15-octadecadienoic acid	Oleic acid	<LOQ	-	<LOQ	-	<LOQ	-
**Peak**	**Name**	**Equivalents Expressed**	**UTC_C**		**AC_C**		**WD_C**	
			Concentration * µg g^−1^ dw	**Precision ^#^ (RSD, %)**	Concentration * µg g^−1^ dw	**Precision ^#^ (RSD, %)**	Concentration * µg g^−1^ dw	**Precision ^#^ (RSD, %)**
2	Monogalloyl-glucose	Gallic acid	<LOQ ^c^	-	0.43 ± 0.01 ^b^	3.46%	0.78 ± 0.03 ^a^	3.94%
3	Pedunculagin (bis-HHDP-glucose)	Gallic acid	1.45 ± 0.05 ^b^	3.52%	3.71 ± 0.15 ^a^	4.18%	0.96 ± 0.05 ^c^	5.46%
5	Pedunculagin (bis-HHDP-glucose)	Gallic acid	1.11 ± 0.03 ^b^	2.39%	10.08 ± 0.56 ^a^	5.51%	0.65 ± 0.05 ^b^	6.99%
6	Gallic acid methylester	Gallic acid	1.37 ± 0.04 ^b^	2.59%	3.75 ± 0.03 ^a^	0.86%	0.34 ± 0.01 ^c^	4.14%
8	(+)Catechin	Catechin	0.47 ± 0.01 ^b^	1.60%	0.66 ± 0.01 ^a^	2.18%	0.3 ± 0.01 ^c^	1.68%
9	Dicarboxylic acid derivative 2	Ellagic acid	<LOQ	-	<LOQ	-	<LOQ	-
10	Dicarboxylic acid derivative 3	Ellagic acid	<LOQ	-	<LOQ	-	<LOQ	-
11	Dihydroxytetralone hexoside	Chlorogenic acid	0.68 ± 0.01 ^a^	0.74%	0.71 ± 0.01 ^a^	1.67%	0.52 ± 0.02 ^b^	3.16%
14	HHDP digalloyl glucose	Gallic acid	0.15 ± 0.01 ^b^	4.72%	3.97 ± 0.03 ^a^	0.81%	0.08 ± 0.00 ^c^	0.69%
16	Sinapic acid hexose	Sinapic acid	3.95 ± 0.05 ^c^	1.15%	25.1 ± 0.03 ^a^	0.11%	16.59 ± 0.05 ^b^	0.27%
17	p-Coumaric acid derivative 1	4-Coumaric acid	2.4 ± 0.01 ^c^	0.44%	30.03 ± 0.1 ^b^	0.32%	40.04 ± 0.11 ^a^	0.27%
19	Gallic acid derivative 4	Gallic acid	2.18 ± 0.01 ^b^	0.63%	1.88 ± 0.04 ^c^	1.89%	2.44 ± 0.16 ^a^	6.47%
20	Glansreginin B	Ellagic acid	0.68 ± 0.01 ^c^	1.94%	1.57 ± 0.02 ^b^	1.23%	3.46 ± 0.07 ^a^	1.99%
21	Dicarboxylic acid derivative 3	Ellagic acid	<LOQ	-	<LOQ	-	<LOQ	-
22	Dicarboxylic acid derivative 3	Ellagic acid	<LOQ	-	<LOQ	-	<LOQ	-
23	Ellagic acid pentoside	Ellagic acid	0.41 ± 0.01 ^c^	2.58%	3.46 ± 0.07 ^a^	1.97%	0.65 ± 0.01 ^b^	1.39%
24	Dicarboxylic acid derivative 3	Ellagic acid	0.72 ± 0.03 ^c^	3.86%	1.57 ± 0.02 ^b^	1.19%	3.55 ± 0.24 ^a^	6.66%
25	Ellagic acid	Ellagic acid	7.62 ± 0.24 ^c^	3.15%	17.78 ± 0.14 ^a^	0.78%	10.06 ± 0.35 ^b^	3.45%
27	Glansreginin A	Ellagic acid	34.76 ± 0.33 ^c^	0.96%	39.9 ± 0.74 ^b^	1.86%	58.22 ± 1.17 ^a^	2.01%
29	Glansreginin A	Ellagic acid	<LOQ	-	0.83 ± 0.00 ^a^	0.42%	0.2 ± 0.01 ^b^	4.58%
30	Glansreginin A	Ellagic acid	<LOQ	-	1.02 ± 0.01 ^a^	0.87%	0.95 ± 0.07 ^a^	7.72%
35	3-p-Coumaroylquinic acid	4-Coumaric acid	<LOQ	-	0.87 ± 0.01 ^a^	1.00%	0.87 ± 0.05 ^a^	5.80%
36	5-O-(3′-O-Glucosylcaffeoyl)quinic acid	Chlorogenic acid	1.07 ± 0.03 ^a^	3.13%	0.85 ± 0.02 ^b^	2.70%	0.83 ± 0.03 ^b^	3.82%
40	9,12,13-trihydroxy-10,15-octadecadienoic acid	Oleic acid	<LOQ	-	<LOQ	-	<LOQ	-
42	9,12,13-trihydroxy-10-octadecenoic acid	Oleic acid	2.37 ± 0.11 ^b^	4.66%	1.52 ± 0.03 ^c^	1.84%	2.61 ± 0.08 ^a^	2.96%
43	9,12,13-trihydroxy-10-octadecenoic acid	Oleic acid	<LOQ	-	0.91 ± 0.01 ^a^	1.04%	0.99 ± 0.04 ^a^	3.77%
44	9,12,13-trihydroxy-10,15-octadecadienoic acid	Oleic acid	<LOQ	-	<LOQ	-	<LOQ	-

* Each value represents mean ± SD (*n* = 3); Means followed by different letters are significantly different according to Tukey’s honestly significant difference (HSD). Means followed by the same letter are not significantly different; ^#^ RSD, relative standard deviation (SD × 100/Mean).

**Table 5:** Resonance assignments with chemical shifts in identified metabolites.

Molecule	^1^H Shift	Multiplicity	Assignment	UTC_A	AC_A	WD_A	UTC_B	AC_B	WD_B	UTC_C	AC_C	WD_C
Fatty acids (terminal -CH_3_)	0.82	t	**-CH_3_**	-	-	+	+	+	+	+	+	+
Isoleucine	0.94	t	-CH_3_	-	-	-	+	+	+	+	+	+
Leucine	0.97	t	-CH_3_	+	+	+	-	+	+	+	-	+
Valine	1.05	d	-CH_2_	+	+	+	+	+	+	+	+	+
Fatty acids (methylene)	1.23		-**CH_2_**_n_	-	-	+	+	+	+	+	+	+
Threonine	1.33	d	-CH_2_	+	+	+	+	+	+	+	+	+
Alanine	1.48	d	-CH_2_	+	+	+	+	+	+	+	+	+
Arginine	1.62–1.78	m	n.a.	+	+	+	+	+	+	+	+	+
Acetate	1.92	s	-CH_3_	+	+	+	+	+	+	+	+	+
Fatty acids (allylic)	1.97		-**CH_2_**-CH=CH	-	-	+	+	+	+	+	+	+
Fatty acids (acyl chains)	2.24		-**CH_2_**-COO	-	-	+	+	+	+	+	+	+
Sucrose	4.07	dd	-CHOH	-	-	+	-	+	+	+	+	+
Malate	4.22	dd	-CHOH	+	+	+	+	+	+	+	+	+
Fatty acids (olefinic portions)	5.28	m	**CH**=**CH**	-	-	+	+	+	+	+	+	+

## Data Availability

Data will be made available on request.

## Supplementary Materials

The following supporting information can be downloaded at: https://www.mdpi.com/article/10.3390/antiox14091079/s1, Figure S1: TIC chromatograms of *Juglans regia* L. extracts; Table S1: LC-MS method validation parameters; Table S2: Putative identification of compounds; Figure S2: Representative ^1^H NMR of extracts.

## Abbreviations

The following abbreviations are used in this manuscript: DMEM: Dulbecco’s Modified Eagle Medium; EMT: epithelial–mesenchymal transition; FAAs: free amino acids; FBS: fetal bovine serum; GAE: gallic acid equivalents; IL-1β: interleukin-1-beta; LOD: limits of detection; LOQ: limits of quantification; QE: quercetin equivalents; RSD: Relative Standard Deviation; SD: standard deviation; TCA: trichloroacetic acid; TFC: total flavonoid content; TGF-β: transforming growth factor beta; TNF-α: tumor necrosis factor alpha; TPC: total phenolic content; VEGF: vascular-endothelial growth factor; WD: wood distillate; XIC: Extracted ion chromatograms; XRF: X-ray fluorescence.
