# Supplementary material for: Unveiling Wound Healing Properties of Biostimulated Walnut Kernel Extracts via Epithelial Mesenchymal Transition: Switching a Nutritional Matrix into a Therapeutic Remedy
Source: Antioxidants (Basel). 2025 Sep 2;14(9):1079. doi: 10.3390/antiox14091079 (PMC12466580; doi:10.3390/antiox14091079)

SUPPLEMENTARY

Unveiling Wound Healing Properties of Biostimulated Walnut Kernel Extracts via Epithelial Mesenchymal Transition: Switching a Nutritional Matrix into a Therapeutic Remedy

Riccardo Fedeli [1,†], Elia Ranzato [2,†], Simona Martinotti [2], Manuela Giovanna Basilicata [3], Ludovica Marotta [4], Marianna Fava [4], Ilaria Cursaro [4], Giulio Tremori [1], Gregorio Bonsignore [2], Gabriele Carullo [1,4,*], Sandra Gemma [1,4,*], Giovanna Aquino [5], Pietro Campiglia [6], Giacomo Pepe [6,7], Stefania Butini [1,4], Stefano Loppi [1,7,‡] and Giuseppe Campiani [1,4,‡]

[1] BioAgry Lab, Department of Life Sciences, University of Siena, 53100 Siena, Italy; riccardo.fedeli@unisi.it (R.F.); giulio.tremori@student.unisi.it (G.T.); butini3@unisi.it (S.B.); stefano.loppi@unisi.it (S.L.); giuseppe.campiani@unisi.it (G.C.)
[2] Department of Science and Technological Innovation, University of Piemonte Orientale, Viale Teresa Michel 11, 15121 Alessandria, Italy; elia.ranzato@uniupo.it (E.R.); simona.martinotti@uniupo.it (S.M.); gregorio.bonsignore@uniupo.it (G.B.)
[3] Department of Advanced Medical and Surgical Sciences, University of Campania "Luigi Vanvitelli", 80138 Naples, Italy; manuelagiovanna.basilicata@unicampania.it or mbasilicata@unisa.it
[4] TheraFood Research, Department of Biotechnology, Chemistry and Pharmacy, University of Siena, 53100 Siena, Italy; ludovica.marotta@student.unisi.it (L.M.); marianna.fava@student.unisi.it (M.F.); ilaria.cursaro@student.unisi.it (I.C.)
[5] AREA Science Park, Laboratorio di Multi-omica Area Sud (LAAS), 84081 Baronissi, Italy; gaquino@unisa.it
[6] Department of Pharmacy, University of Salerno, 84084 Fisciano, Italy; pcampiglia@unisa.it (P.C.); gipepe@unisa.it (G.P.)
[7] NBFC, National Biodiversity Future Center, 90133 Palermo, Italy
* Correspondence: gabriele.carullo@unisi.it (G.C.); gemma@unisi.it (S.G.)
† These authors contributed equally to this work.
‡ Co-last and senior authors.

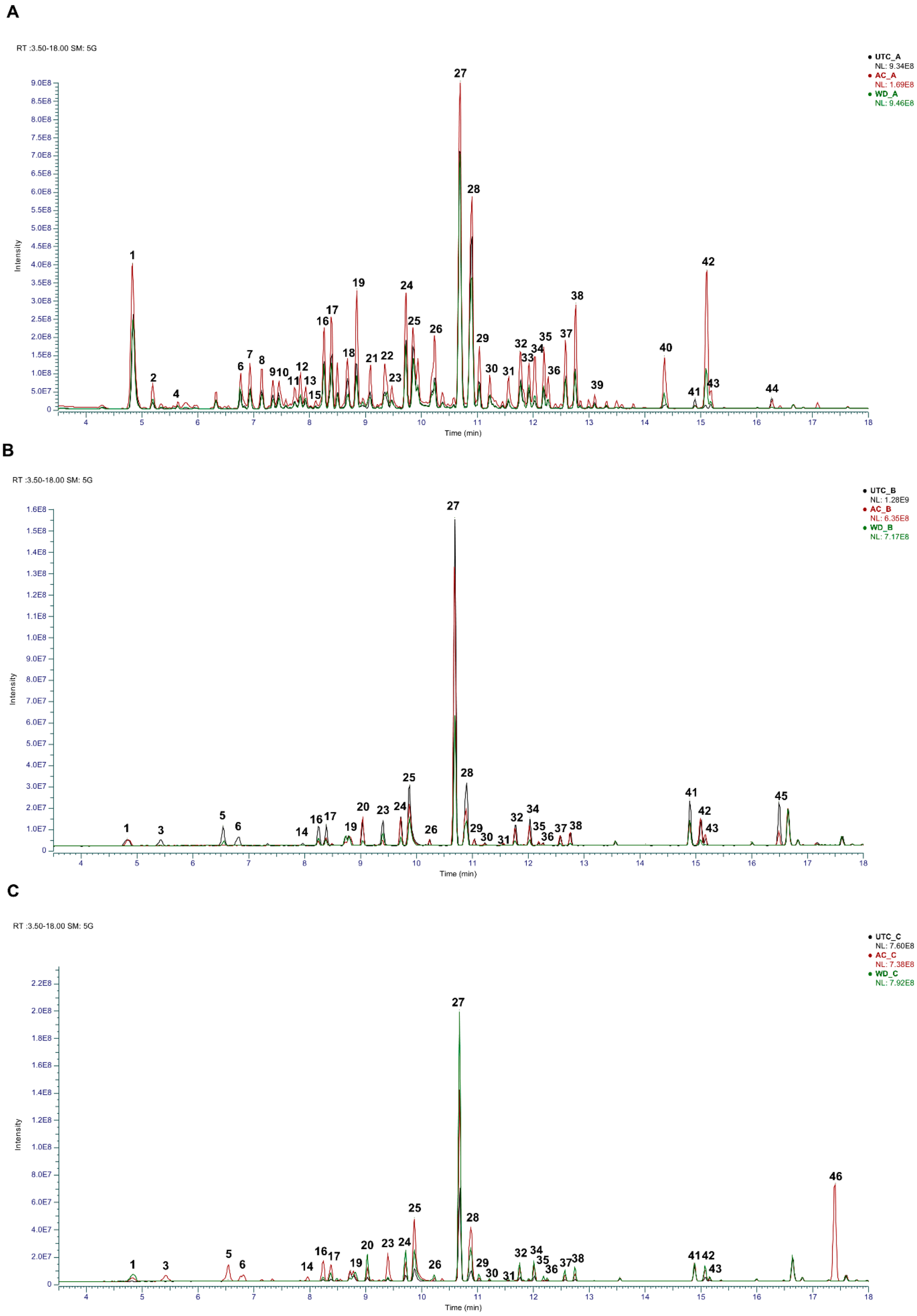

Figure S1. TIC chromatogram of *Juglans regia* L. water extract (**A**), acetone extract (**B**) and Folch green extract (**C**) acquired in negative ionization mode.

Table S1. LC-MS method validation parameters.

Compound	RT (min) ± SD (*n* = 4)	Linear range (μg mL^{-1})	Regression equation	Correlation coefficient (R^2)	LOQ (μg mL^{-1})	LOD (μg mL^{-1})		QC levels	Nominal concentration (μg mL^{-1})	Mean ± SD (μg mL^{-1})	Precision (RSD.%)	Accuracy (%)
**Gallic acid**	0.707 ± 0.004	0.1-25	y = 4.31E-08x -1.67E-02	0.9996	0.005	0.002	*Intra-day*	LQC	1	0.89 ± 0.06	6.40%	112.95%
								MQC	2	2.09 ± 0.19	9.13%	95.73%
								HQC	5	5.24 ± 0.11	2.02%	95.38%
			y = 4.90E-08x -3.49E-01	0.9991			*Inter-day*	LQC	1	0.96 ± 0.01	1.27%	103.87%
								MQC	2	2.12 ± 0.04	1.67%	94.42%
								HQC	5	4.90 ± 0.06	1.21%	102.10%
**Sinapic acid**	8.94 ± 0.01	1-200	y = 9.18E-07x + 6.68E-01	0.9994	0.032	0.010	*Intra-day*	LQC	5	5.40 ± 0.01	0.17%	92.60%
								MQC	10	9.72 ± 0.20	2.04%	102.92%
								HQC	25	23.2 ± 0.13	0.58%	107.74%
			y = 9.24E-07x + 7.94E-01	0.9998			*Inter-day*	LQC	5	5.46 ± 0.11	2.02%	91.54%
								MQC	10	9.73 ± 0.13	1.35%	102.76%
								HQC	25	22.44 ± 0.35	1.57%	111.40%
**Ellagic acid**	9.40 ± 0.01	1-100	y = 3.34E-08x -7.82E-01	0.9998	0.135	0.044	*Intra-day*	LQC	5	5.40 ± 0.11	2.06%	92.62%
								MQC	10	10.95 ± 0.13	1.18%	91.33%
								HQC	25	24.54 ± 0.09	0.36%	101.88%
			y = 3.28E-08x -1.85E-01	0.9996			*Inter-day*	LQC	5	5.59 ± 0.09	1.57%	89.44%
								MQC	10	10.61 ± 0.17	1.56%	94.22%
								HQC	25	23.46 ± 0.06	0.26%	106.56%
**Chlorogenic acid**	6.68 ± 0.01	0.1-25	y = 4.39E-08x + 9.77E-02	0.9999	0.005	0.002	*Intra-day*	LQC	1	0.98 ± 0.02	1.82%	102.29%
								MQC	2	2.03 ± 0.14	6.76%	98.40%
								HQC	5	5.00 ± 0.11	2.26%	100.01%
			y = 4.78E-08x + 8.98E-02	0.9999			*Inter-day*	LQC	1	0.99 ± 0.01	1.41%	101.42%
								MQC	2	1.93 ± 0.04	2.13%	103.72%
								HQC	5	4.93 ± 0.10	1.98%	101.40%
***p*-Coumaric acid**	7.76 ± 0.01	0.05-10	y = 3.83E-08x -7.54E-02	0.9998	0.004	0.001	*Intra-day*	LQC	0,5	0.52 ± 0.01	1.75%	95.63%
								MQC	1	1.11 ± 0.01	1.14%	90.49%
								HQC	5	5.02 ± 0.04	0.76%	99.69%
			y = 3.81E-08x -7.76E-02	0.9995			*Inter-day*	LQC	0,5	0.50 ± 0.01	1.82%	100.40%
								MQC	1	1.06 ± 0.04	4.04%	94.32%
								HQC	5	5.14 ± 0.06	1.08%	97.26%
**Catechin**	6.22 ± 0.01	0.1-10	y = 3.71E-08x + 6.11E-02	0.9999	0.013	0.004	*Intra-day*	LQC	1	1.02 ± 0.04	4.19%	97.92%
								MQC	2	2.00 ± 0.07	3.32%	99.80%
								HQC	5	5.00 ± 0.26	5.16%	100.01%

			y = 3.45E-08x - 1.51E-02	0.9998			*Inter-day*	LQC	1	1.063 ± 0.003	0.29%	94.05%
								MQC	2	1.97 ± 0.05	2.72%	101.51%
								HQC	5	4.93 ± 0.02	0.32%	101.35%
**Oleic acid**	21.04 ± 0.02	0.5-200	y = 4.39E-06x -3.08E+00	0.9997	0.718	0.237	*Intra-day*	LQC	10	11.59 ± 0.15	1.31%	86.29%
								MQC	25	25.16 ± 0.99	3.93%	99.35%
								HQC	50	51.26 ± 1.54	3.01%	97.55%
			y = 3.97E-06x -2.71E+00	0.9995			*Inter-day*	LQC	10	10.66 ± 0.18	1.68%	93.78%
								MQC	25	25.48 ± 1.15	4.51%	98.13%
								HQC	50	48.49 ± 1.76	3.64%	103.11%

* Each value represents mean ± SD (n = 3);
# RSD, relative standard deviation (SD × 100/Mean);
LQC: low quality control; MQC: medium quality control; HQC: high quality control.

Table S2. Putatively identified compounds from *Juglans regia* L. extracts by RP-UHPLC-ESI-Orbitrap-MS/MS analysis.

Peak	Rt (min)	Name	Formula	MW	m/z	MS2	Error (ppm)	Adduct	MSI Level*	Equivalents expressed
1	4.85	UNK1	$C_{16}H_{19}NO_9$	369.1054	368.0980	144.0456; 89.0245; 59.0139; 119.0351; 162.0558	-1.62	$[M-H]^{-1}$	4	-
2	5.20	Monogalloyl-glucose	$C_{13}H_{16}O_{10}$	332.0745	331.0672	169.0100	0.39	$[M-H]^{-1}$	2	Gallic acid
3	5.43	Pedunculagin (bis-HHDP-glucose)	$C_{34}H_{24}O_{22}$	784.0758	783.0686	300.9989; 275.0198;249.0403; 481.0615; 257.0083	-0.04	$[M-H]^{-1}$	2	Gallic acid
4	5.65	Vanillic acid 4-β-D-glucopyranoside	$C_{14}H_{18}O_9$	330.0952	329.0879	167.0349; 123.0452; 152.0115; 108.0217	0.3	$[M-H]^{-1}$	2	-
5	6.55	Pedunculagin (bis-HHDP-glucose)	$C_{34}H_{24}O_{22}$	784.0758	783.0686	300.9989; 275.0198;249.0403; 481.0615; 257.0083	-0.07	$[M-H]^{-1}$	2	Gallic acid
6	6.82	Gallic acid methylester	$C_8H_8O_5$	184.0379	183.02992	124.0166; 168.0065; 94.9168	0.11	$[M-H]^{-1}$	2	Gallic acid
7	6.94	UNK2	$C_{14}H_{14}N_2O_6$	306.0851	305.0779	189.0669; 174.9561; 146.0611; 132.0303; 115.0037	-0.42	$[M-H]^{-1}$	4	-
8	7.18	(+) Catechin	$C_{15}H_{14}O_6$	290.0791	289.0719	245.0830; 231.0290; 203.0714; 174.9570; 158.9788; 125.0249; 179.0343	0.32	$[M-H]^{-1}$	1	Catechin
9	7.35	Dicarboxylic acid derivative 2	$C_{12}H_{20}O_6$	260.1260	259.1187	73.0295; 159.0663; 85.0295; 141.0921; 241.1084	-0.03	$[M-H]^{-1}$	2	Ellagic acid
10	7.45	Dicarboxylic acid derivative 3	$C_{12}H_{20}O_6$	260.1260	259.1187	73.0295; 159.0663; 85.0295; 141.0921; 241.1084	0.08	$[M-H]^{-1}$	2	Ellagic acid
11	7.73	Dihydroxytetralone hexoside	$C_{16}H_{20}O_8$	340.1153	339.1080	159.0452; 177.0559	-1.58	$[M-H]^{-1}$	2	Chlorogenic acid
12	7.84	UNK3	$C_{18}H_{28}O_{11}$	420.1627	419.1555	177.0922; 133.1024; 195.1029; 221.0821; 89.0245	-1.04	$[M-H]^{-1}$	4	-
13	7.93	Benzyl alcohol hexose pentose	$C_{18}H_{26}O_{10}$	402.1522	401.1449	269.1031; 161.0456; 131.0349; 125.0246; 101.0244; 191.0560; 113.0244; 71.0139	-0.99	$[M-H]^{-1}$	2	-
14	7.97	HHDP digalloyl glucose	$C_{35}H_{22}N_4O_{18}$	786.0916	785.0845	300.990; 419.0623; 633.0739; 615.0634; 275.0198	-1.61	$[M-H]^{-1}$	2	Gallic acid
15	8.10	Regiolone	$C_{10}H_{10}O_3$	178.0631	177.0558	159.0452; 175.0402; 131.0503; 93.0349; 115.0554	0.5	$[M-H]^{-1}$	2	-
16	8.26	Sinapic acid hexose	$C_{18}H_{25}O_{12}$	386.1936	431.1918	153.0923; 101.0245; 89.0244; 71.0139; 205.1234; 223.1341; 161.0456; 385.1845;	-1.32	$[M+FA-H]^{-1}$	2	Sinapic acid
17	8.40	p-Coumaric acid derivative 1	$C_{19}H_{32}O_8$	388.2092	433.2074	179.0566; 119.0349; 207.1385; 163.0500	-1.32	$[M+FA-H]^{-1}$	2	4-Coumaric acid
18	8.68	L-N-(1H-Indol-3-ylacetyl) aspartic acid	$C_{14}H_{14}N_2O_5$	290.0902	289.0830	88.0404; 132.0302; 173.0721; 115.0037; 245.0932	-0.32	$[M-H]^{-1}$	2	-
19	8.84	Gallic acid derivative 4	$C_{12}H_{22}O_6$	262.1416	261.1343	201.1133; 187.0977; 199.1341; 243.1239; 169.0871; 225.1128; 125.0973	-0.35	$[M-H]^{-1}$	2	Gallic acid

20	9.04	Glansreginin B	$C_{24}H_{38}O_{15}$	566.2211	565.2139	241.1082; 343.1393; 197.1184; 403.1606	0.2	$[M-H]^{-1}$	2	Ellagic acid
21	9.09	Dicarboxylic acid derivative 3	$C_{18}H_{28}O_{10}$	404.1677	403.1604	241.1082; 283.1188; 343.1393; 197.1184	-1.37	$[M-H]^{-1}$	2	Ellagic acid
22	9.35	Dicarboxylic acid derivative 3	$C_{18}H_{28}O_{10}$	404.1676	403.1603	241.1082; 283.1188; 343.1393; 197.1184	-1.72	$[M-H]^{-1}$	2	Ellagic acid
23	9.40	Ellagic acid pentoside	$C_{19}H_{14}O_{12}$	434.0481	433.0409	300.9989; 228.9530	-0.84	$[M-H]^{-1}$	2	Ellagic acid
24	9.728	Dicarboxylic acid derivative 3	$C_{18}H_{28}O_{10}$	404.1678	403.1604	241.1082; 283.1188; 343.1393; 197.1184	-1.18	$[M-H]^{-1}$	2	Ellagic acid
25	9.86	Ellagic acid	$C_{14}H_{6}O_{8}$	302.0062	300.9989	257.0090; 229.0143	-0.35	$[M-H]^{-1}$	1	Ellagic acid
26	10.24	UNK4	$C_{28}H_{39}NO_{14}$	613.2364	612.2295	261.1343; 187.0976; 303.1451	-0.87	$[M-H]^{-1}$	4	-
27	10.69	Glansreginin A	$C_{28}H_{35}NO_{13}$	593.2102	592.2033	197.1184; 241.1082; 343.1394; 403.1591	-1.13	$[M-H]^{-1}$	2	Ellagic acid
28	10.90	Blumenol C O-glucoside	$C_{19}H_{32}O_{7}$	372.2141	417.2123	161.0455; 101.0243; 71.0142; 89.0247; 59.0140	-1.9	$[M+FA-H]^{-1}$	3	-
29	11.04	Glansreginin A	$C_{28}H_{35}NO_{13}$	593.2107	592.2036	197.1184; 241.1082; 343.1394; 403.1605	-0.2	$[M-H]^{-1}$	2	Ellagic acid
30	11.23	Glansreginin A	$C_{28}H_{35}NO_{13}$	593.2107	592.2036	197.1184; 241.1082; 343.1393; 403.1618	-0.25	$[M-H]^{-1}$	2	Ellagic acid
31	11.56	UNK5	$C_{25}H_{32}O_{14}$	556.1789	555.1717	169.0142; 125.0244; 59.0139; 101.0248; 223.0975	-0.54	$[M-H]^{-1}$	4	-
32	11.78	(4R,5S,7R,11x)-11,12-Dihydroxy-1(10)-spirovetiven-2-one 12-glucoside	$C_{21}H_{34}O_{8}$	414.2247	413.2174	59.0138; 71.0139; 89.0247; 101.0244;	-1.54	$[M-H]^{-1}$	2	-
33	11.93	UNK6	$C_{28}H_{31}NO_{12}$	573.1845	572.1774	144.0456; 177.0922; 221.0821; 263.0927	-0.31	$[M-H]^{-1}$	4	-
34	12.04	(4R,5S,7R,11x)-11,12-Dihydroxy-1(10)-spirovetiven-2-one 12-glucoside	$C_{21}H_{34}O_{8}$	414.2251	413.2178	59.0138; 71.0139; 89.0247; 101.0244;	-0.71	$[M-H]^{-1}$	2	-
35	12.20	3-p-Coumaroylquinic acid	$C_{16}H_{18}O_{8}$	338.1002	337.0929	163.0401; 191.9469; 119.0503	0.15	$[M-H]^{-1}$	2	4-Coumaric acid
36	12.27	5-O-(3'-O-Glucosylcaffeoyl) quinic acid	$C_{25}H_{24}O_{12}$	516.2567	515.2497	161.0456; 353.1965; 59.0139; 89.0248	-0.68	$[M-H]^{-1}$	2	Chlorogenic acid
37	12.58	UNK7	$C_{28}H_{33}NO_{12}$	575.2001	574.1931	179.1079; 144.0457; 193.1236; 221.1184	-0.39	$[M-H]^{-1}$	4	-
38	12.76	UNK18	$C_{27}H_{36}N_{10}O_{8}$	628.2726	627.2655	197.1184; 241.1083; 283.1189; 343.1394	1.32	$[M-H]^{-1}$	4	-
39	13.11	UNK9 isomer	$C_{28}H_{31}NO_{12}$	573.1844	572.1775	144.0456; 177.0921; 221.0820; 263.0926	-0.32	$[M-H]^{-1}$	4	-
40	14.35	9,12,13-trihydroxy-10,15 octadecadienoic acid	$C_{18}H_{32}O_{5}$	328.2250	327.2178	211.1340; 229.1445; 291.1967; 171.1027	0.17	$[M-H]^{-1}$	2	Oleic acid
41	14.90	N-Undecanoylglycine	$C_{13}H_{25}NO_{3}$	243.1834	242.1762	224.160; 174.9561; 74.6074; 198.1870	4.39	$[M-H]^{-1}$	2	-
42	15.10	9,12,13-trihydroxy-10- octadecenoic acid	$C_{18}H_{34}O_{5}$	330.2406	329.2334	211.1340; 229.1446; 171.1030	0.16	$[M-H]^{-1}$	2	Oleic acid
43	15.19	9,12,13-trihydroxy-10- octadecenoic acid	$C_{18}H_{34}O_{5}$	330.2406	329.2334	211.1340; 229.1445; 171.1027	0.03	$[M-H]^{-1}$	2	Oleic acid
44	16.28	9,12,13-trihydroxy-10,15 octadecadienoic acid	$C_{18}H_{32}O_{5}$	328.2250	327.2177	309.2076; 291.1963; 221.1191; 229.0921	-0.03	$[M-H]^{-1}$	2	Oleic acid
45	16.50	Macelignan	$C_{20}H_{24}O_{4}$	328.1677	327.1602	327.1603; 312.1368;	0.04	$[M-H]^{-1}$	2	-

46	17.40	UNK10	$C_{17}H_{19}N_3O_6$	361.1267	360.1194	311.1302; 283.1031; 241.0862; 146.9390 227.0337; 284.1042; 256.1091; 238.0991	-1.84	$[M-H]^{-1}$	4	-

Figure S2. WD_C spectrum

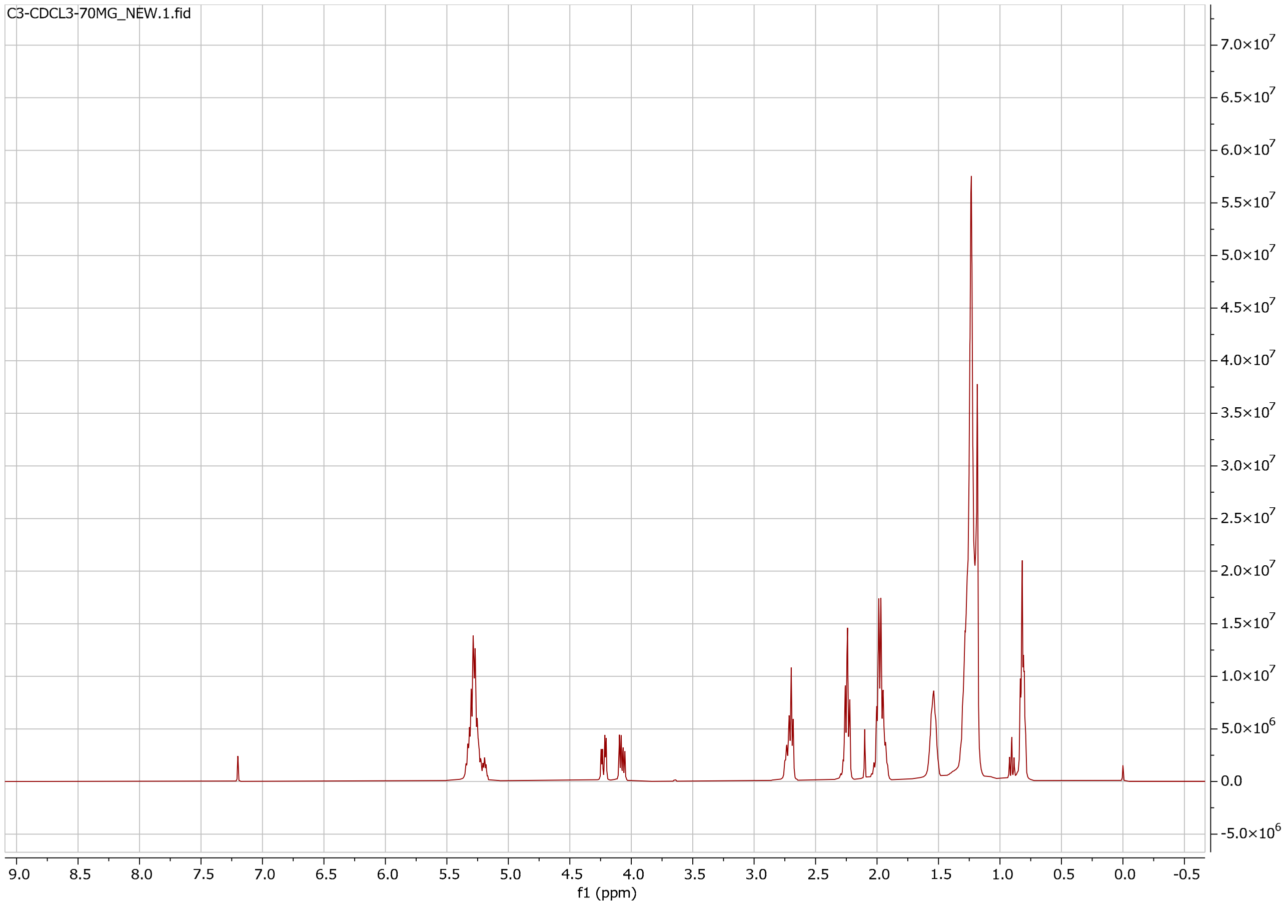

Supplement: Supplementary file 1 [file antioxidants-14-01079-s001.zip › antioxidants-3829906-supplementary.pdf]
